# Electrostatic Interaction Tailored Anion-Rich Solvation Sheath Stabilizing High-Voltage Lithium Metal Batteries

**DOI:** 10.1007/s40820-022-00896-4

**Published:** 2022-07-21

**Authors:** Junru Wu, Ziyao Gao, Yao Wang, Xu Yang, Qi Liu, Dong Zhou, Xianshu Wang, Feiyu Kang, Baohua Li

**Affiliations:** 1 Tsinghua Shenzhen International Graduate School, Tsinghua University, Shenzhen, 518055 People’s Republic of China; 2 School of Materials Science and Engineering, Tsinghua University, Beijing, 100084 People’s Republic of China; 3 College of Materials Science and Engineering, Hunan University, Changsha, 410082 People’s Republic of China

**Keywords:** Electrostatic interaction, Anion-rich solvation sheath, High voltage, Lithium metal batteries, Wide temperature range

## Abstract

Through tailoring interfacial chemistry, electrolyte engineering is a facile yet effective strategy for high-performance lithium (Li) metal batteries, where the solvation structure is critical for interfacial chemistry. Herein, the effect of electrostatic interaction on regulating an anion-rich solvation is firstly proposed. The moderate electrostatic interaction between anion and solvent promotes anion to enter the solvation sheath, inducing stable solid electrolyte interphase with fast Li^+^ transport kinetics on the anode. This as-designed electrolyte exhibits excellent compatibility with Li metal anode (a Li deposition/stripping Coulombic efficiency of 99.3%) and high-voltage LiCoO_2_ cathode. Consequently, the 50 μm-thin Li||high-loading LiCoO_2_ cells achieve significantly improved cycling performance under stringent conditions of high voltage over 4.5 V, lean electrolyte, and wide temperature range (− 20 to 60 °C). This work inspires a groundbreaking strategy to manipulate the solvation structure through regulating the interactions of solvent and anion for high-performance Li metal batteries.

**Supplementary Information:**

The online version contains supplementary material available at 10.1007/s40820-022-00896-4.

## Highlights

- The effect of electrostatic interaction on regulating an anion-rich solvation in electrolyte is firstly proposed.
- The moderate electrostatic interaction between anion and solvent promotes anion-rich solvation sheath, inducing a stable electrolyte|electrode interface with fast Li^+^ transport kinetics.
- The outstanding electrochemical performance of 50 μm-thin Li||high-loading LiCoO_2_ batteries is achieved at high voltage of 4.5 V (even up to 4.6 V), lean electrolyte of 15 μL, and wide temperature range of − 20 to 60 °C.

**Supplementary Information:**

The online version contains supplementary material available at 10.1007/s40820-022-00896-4.

## Graphical abstract

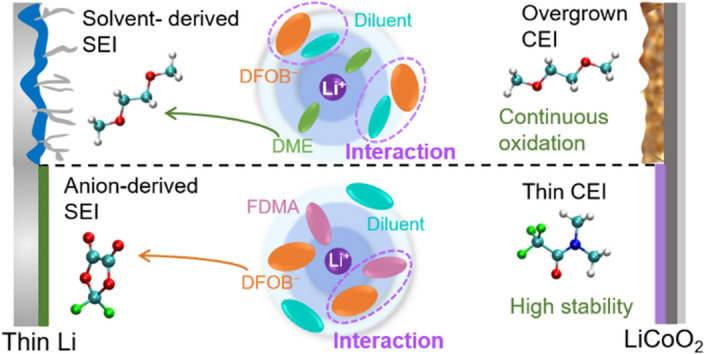

## Introduction

Lithium (Li) metal, with a low mass density (0.534 g cm^−2^), a high theoretical specific capacity (3860 mAh g^−1^), and an extremely low redox potential (− 3.04 V versus standard hydrogen electrode), has been regarded as one of the most prominent anode candidates for next-generation batteries with high energy density [1]. Li metal batteries (LMBs), replacing the widely used graphite anode in Li-ion batteries (LIBs) with Li metal anode, break through the current bottleneck of energy density and significantly improve the value to > 400 Wh Kg^−1^ for future developments [2, 3]. However, the practical application of LMBs is hampered by the uncontrollable dendritic Li growth, the irreversibility of Li plating/stripping, and the interfacial incompatibility. It is well-recognized that the formation of a robust and homogeneous solid electrolyte interphase (SEI) on Li metal anodes can address these above issues, and thus prolonging the lifespan of LMBs [4].

The structure and composition of SEI predominantly depends on the Li^+^ solvation sheath in electrolytes, which are derived from the microscopic interaction between Li^+^ and solvent molecule or anion. In the traditional carbonate-based electrolytes, Li^+^ are mostly coordinated with the carbonate solvent molecule to create a solvent-separated ion pair (SSIP) structure, which induces an organics-rich interface and leads to poor performances for LMBs [5, 6]. Recently, it is reported that a unique solvation shell enriched with anion aggregations can induce the formation of anion-derived SEI, which is beneficial for stabilizing Li anodes [5, 7]. Until now, various strategies have been developed to construct the anion-containing solvation sheath via electrolyte component design for improving electrochemical performances of LMBs. The first proposed highly concentrated electrolytes (HCEs), which has been proved to be effective in enhancing the interactions between Li^+^ and anions by increasing the salt-to-solvent ratio and therefore, leading to the occupation of anions at the inner solvation sheath [8]. Subsequently, localized high-concentrated electrolytes (LHCEs) have been developed by introducing the hydrofluoroether as diluents into HCEs, which could also maintain an anion-participated solvation structure similar to that of HCEs [9, 10]. In addition, the employment of a low-polarity solvent with weak solvating ability could allow more anions to coordinate with Li^+^ for the generation of ion pairs (namely, weakly solvating electrolyte, WSE) [11, 12]. This single-solvent electrolyte system was further expanded by Bao’s group, who designed a single solvent by the incorporation of –CF_2_– group in 1,4-dimethoxybutane to solvate Li^+^ with a peculiar Li–F coordination, contributing to a high anion/solvent ratio in the solvation sheath [13]. Following this work, they further demonstrated that strengthening steric hindrance effect could also facilitate the prevalence of ion pairs and aggregates in the solvation structure via reducing the dissolution ability of polar solvent [14]. Meanwhile, some essential approaches toward dual-anion solvation structure involves promoting the dissociation of Li nitrate (LiNO_3_) by anion receptor addition and solvent formula [6, 15, 16], where NO_3_^−^ could directly participate the Li^+^ solvation sheath. Despite these achievements on anions-regulated solvation of electrolyte, the detailed relationship between anions and solvents/diluents are still unclear, and the mechanism by which this interaction affects the solvation structure remains ambiguous.

Herein, we creatively propose an electrostatic interaction between anion and solvent to regulate the solvation sheath of electrolyte systems. A novel electrolyte containing 1 M Li difluoro(oxalato)borate (LiDFOB) in the mixture of 2,2,2-trifluoro-N, N-dimethylacetamide (FDMA) and 1,1,2,3,3,3-hexafluoropropyl-2,2,2-trifluoroethylether (HTE) (1: 1 by volume) (denoted as “DFH”) is designed, where the moderate interaction between DFOB^−^ anion and FDMA solvents facilitates the construction of anion-rich solvation sheath, as demonstrated by the calculations and characterizations. Benefiting from the formation of high-quality SEI derived from this unique solvation, both Li||Li symmetric and Li||Cu cells exhibit high reversibility of Li plating/stripping behavior, as well as compact Li deposition and fast Li^+^ transport kinetics. Furthermore, LMBs with 50 μm Li foil and high-mass loading LiCoO_2_ cathode exhibit excellent electrochemical performances under high voltage, lean electrolyte, and wide temperatures range. This work sheds light on an insightful principle underlying the interaction between anion and solvent for regulating solvation of electrolytes, providing a brand-new perspective for electrolyte design employment.

## Experimental and Calculation

### Preparation and Characterization of Electrolytes

The lithium salts including lithium hexafluorophosphate salt (LiPF_6_, 99.99%), LiDFOB (99.8%) were purchased from Capchem, China. Ethylene carbonate (EC, DoDoChem, 99.98%), ethyl methyl carbonate (EMC, DoDoChem, 99.9%), dimethyl ether (DME, DoDoChem, 99.95%), FDMA (TCI, 98%) and HTE (J&K Scientific Ltd, 97%) were dried with a 4 Å molecular sieve (Sigma-Aldrich) before use. Molarity (M, moles of salt in liters of solution (mol L^−1^) are used to denote the salt concentration in electrolytes. These chemicals were kept, and electrolyte preparation was carried out in an Ar-filled glove box (MBraun) with the concentrations of moisture and oxygen below 0.5 ppm.

^1^H nuclear magnetic resonance (NMR) spectra were recorded on a Bruker AVANCEIII400 with chloroform-d6 as solvent. Raman spectra were performed by a miniature laser confocal Raman spectrometer (Horiba LabRAM HR800, France) with a 532 nm laser at room temperature. For the combustion test, a 0.5 g sample of electrolyte was poured into a dish, and then optical photographs and movies were recorded after the sample was ignited. Ionic conductivities of the electrolyte samples were tested by electrochemical impedance spectra (EIS) with a frequency range of 10^5^–1 Hz at an alternating potential amplitude of 5 mV and 6 points per decade on a VMP3 multichannel electrochemical station (Bio Logic Science Instruments, France). The tested cell was assembled by immersing two stainless steel blocking electrodes into the electrolyte sample. The Li-ion transference number (*t*_*Li*_^+^) of the electrolyte samples was measured using the method described by Abraham et al*.* [17]. The procedures were as follows: symmetric Li||Li cell was assembled and then the polarization currents, including the initial (*I*^o^) and steady-state (*I*^ss^) current values, were recorded under a small polarization potential (Δ*V*) of 10 mV. Meanwhile, the initial and steady-state values of the bulk resistances (*R*_b_^o^ and *R*_b_^ss^) and electrode|electrolyte interfacial resistances (*R*_i_^o^ and *R*_i_^ss^) were examined by EISs before and after the potentiostatic polarization. The *t*_*Li*_^+^ was calculated based on the following equation:

1 $$t_{Li}^{ + } = \frac{{I^{ss} (\Delta V - I^{o} R_{i}^{o} )}}{{I^{o} (\Delta V - I^{ss} R_{i}^{ss} )}}$$

The oxidative stability of the electrolyte was investigated by LSV tests on Li||stainless steel cells. The oxidation potential value of the electrolyte was determined as the voltage at which the current was increased to 10 μA.

Galvanostatic cycling measurements consisting of repeated 1 h charge-1 h discharge cycles were conducted in symmetrical Li||Li cells at 0.5 mA cm^−2^ to evaluate the compatibility of electrolytes with Li metal. For activation energy measurements, symmetric Li||Li cells using various electrolytes were cycled 10 times at a current density of 0.5 mA cm^−2^ and then kept under 283, 293, 303, 313, and 323 K to record the temperature-dependent EISs. The EIS measurements were performed at an alternating potential amplitude of 5 mV with 6 points per decade recorded on the VMP3 multichannel electrochemical station with a frequency range of 10^5^ Hz to 1 Hz. By fitting the EISs to the equivalent circuit shown in the inset of Fig. S9, the values of the SEI resistance (*R*_*sei*_) and the ion transfer resistance (*R*_*ct*_) can be obtained **(**Tables S1-S3**)**. The activation energy (*E*_*a*_) is then derived from the Arrhenius equation as follows:

2 $$k = \frac{T}{{R_{res} }} = A\exp \left( { - \frac{Ea}{R}} \right)$$

where *k* represents the rate constant, *T* is the absolute temperature, *R*_*res*_ represents *R*_*ct*_ or *R*_*sei*_, A is the preexponential constant, *E*_*a*_ is the activation energy, and R is the standard gas constant.

Li||Cu coin cells were assembled to study the Coulombic efficiencies of Li depositing/stripping in different electrolytes using the method described by Zhang et al*.* [18]. The Cu substrate was pretreated with a Li plating/stripping cycle with a capacity of 5 mAh cm^−2^ at 0.5 mA cm^−2^. After deposition of 5 mAh cm^−2^ Li reservoir (*Q*_*T*_) onto the Cu substrate at 0.5 mA cm^−2^, the cell was charged-discharged with a capacity of 1 mAh cm^−2^ (*Q*_*C*_) for *n* cycles. Afterward, the residual Na reservoir was ultimately stripped to 1 V at 0.5 mA cm^−2^, and the final stripping charge (*Q*_*S*_) that corresponds to the amount of Li remaining after cycling) was measured. The average CE over *n* cycles can be calculated as:

3 $$CE_{avg} = \frac{{nQ_{C} + Q_{S} }}{{nQ_{C} + Q_{T} }}$$

Cu substrates were obtained from disassembled Li||Cu cells after depositing 2 mAh cm^−2^ of Li at 0.2 mAh cm^−2^ for further field emission scanning electron microscope (FE-SEM, SU8010) characterization. In-depth XPS (PHI 5000 VersaProbe II, in which the thickness values in the XPS depth profiles were estimated from the calibrated sputtering of SiO_2_) and AFM characterization was conducted on Cu foils after 10 cycles of plating/stripping with 1 mAh cm^−2^ Li at 0.2 mA cm^−2^.

### Battery Assembly and Characterization.

The LiCoO_2_ cathode with a LiCoO_2_: Super-P: PVDF weight ratio of 96.4:1.8:1.8 was purchased from Guangdong Canrd New Energy Technology Co., Ltd. The average loading of active material was about 10 mg cm^−2^. CR2032 coin cells were assembled in a high-purity Ar-filled glovebox (O_2_ < 0.5 ppm, H_2_O < 0.5 ppm) using Celgard 2400 (25 μm, PP/PE/PP) as separator and 50 μm Li foils as anodes. The as-developed Li||LiCoO_2_ cells were operated at 0.1C for 3 cycles before long-term cycling in a voltage range of 3.0 to 4.5 V or 4.6 V on a Land 2001 A battery testing system. After the specified cycling tests, the cells were dissembled in an Ar-filled glove box and the obtained samples were repeatedly rinsed with pure DME solvent to remove the residual electrolyte. The observation of cathode surface was implemented on FE-SEM (Hitachi SU8010, Japan) and high-resolution field emission transmission electron microscopy (FE-TEM, FEI Tecnai G2 F30, USA). The air-sensitive electrode samples were transferred into the vacuum chambers for in-depth XPS at inert atmosphere. EISs of cycled cells were examined in frequency of 10^–2^ to 10^5^ Hz with 6 points per decade by applying a potential amplitude of 5 mV.

### Theoretical Calculations

Atomistic molecular dynamics simulations have been performed in the GROMACS [19] (version 2020.6) simulation package using the General Amber force field (GAFF2). The solution systems were built through randomly placing 80 DFOB^−^, 246 HTE molecules and 80 Li^+^ into a cubic box of ~ 5 nm, and then added 346 FDMA and 385 DME molecules into the corresponding systems, respectively. Molecular dynamics simulations under the iso-thermo and iso-baric (NPT) ensemble after thousands of steps of energy minimization were implemented using the nose–hoover thermostat at 298 K and parrinello-rahman pressure coupling method under 1 atm at for 10 ns. A cutoff length of 1.2 nm was implemented for the non-bonded interactions, and the Particle Mesh Ewald method [20] with a Fourier spacing of 0.1 nm was applied for the long-range electrostatic interactions. All covalent bonds with hydrogen atoms were constraint using the LINCS algorithm [21].

## Results and Discussion

In this work, a commercial electrolyte containing 1 M LiPF_6_ dissolved in EC/EMC (3: 7 by volume) mixed solvents was selected as the baseline. Meanwhile, the electrolytes containing 1 M LiDFOB in the DME, FDMA and DME/HTE (1: 1 by volume) were prepared as the control samples and denoted as “LiDFOB-DME”, “LiDFOB-FDMA” and “DDH” electrolytes, respectively. The as-designed DFH electrolyte displays a low-flammability (Fig. S1) and an excellent oxidative stability (Fig. S2) for high-voltage applications of LMBs.

### Investigation of Electrostatic Effect Between Anion and Solvents

**Fig. 1:**
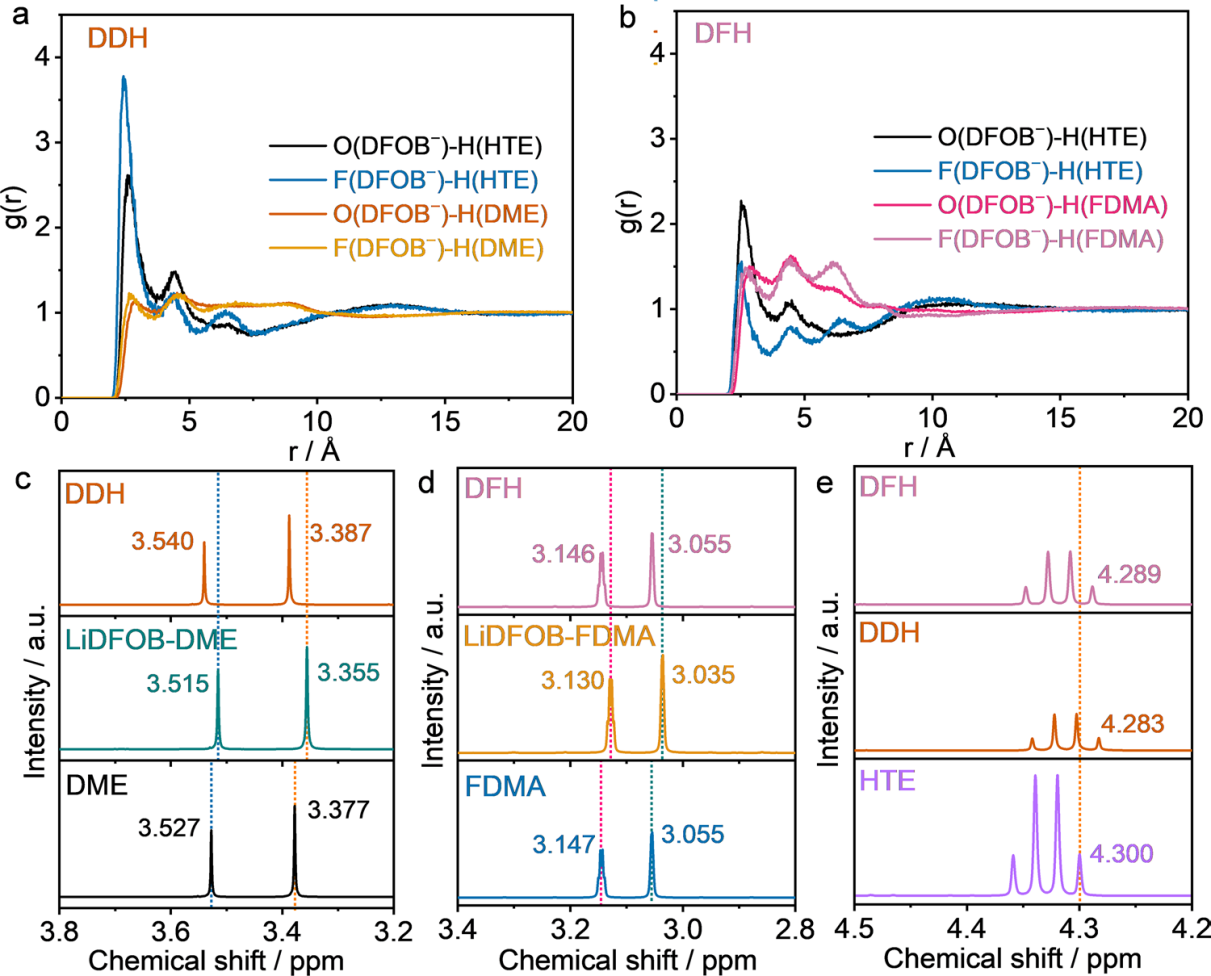
Electrostatic effect between anion and solvents. Radial distribution function between anions and solvent/diluent molecule in **a** DDH and **b** DFH electrolytes. ^1^H NMR spectra of **c** pure DME solvent, LiDFOB-DME and DDH electrolytes, **d** pure FDMA solvent, LiDFOB-FDMA and DFH electrolytes, and **e** pure HTE solvent, DDH and DFH electrolytes

Firstly, molecular dynamics (MD) simulations were performed to investigate the interaction between anions and solvent/diluent molecule. According to the radical distribution function (RDF), the peaks at 2.5 Å is an indication of the existence of electrostatic interaction [22]. It is obvious that in DDH electrolyte, a strong electrostatic interaction between DFOB^−^ anion and H_HTE_ appears while the interaction between DFOB^−^ anion and H_DME_ is rather weak (Fig. 1a). In comparison, DFH electrolyte exhibits electrostatic force with moderate strength between DFOB^−^ anion and H_HTE_/H_FDMA_ (Fig. 1b).

The results from ^1^H NMR spectra of the electrolytes further confirm this kind of electrostatic interaction. As shown in Fig. 1c, after dissolving LiDFOB in pure DME solvent, H_DME_ delivers upfield shifts from 3.527 and 3.377 to 3.515 and 3.355 ppm, respectively, which is probably attributed to the electron-donating properties of DFOB^−^ anion toward H_DME_ [22, 23]. Such a depressed change can be also observed in FDMA solvent with LiDFOB addition (from 3.147 and 3.055 to 3.130 and 3.035 ppm, respectively, Fig. 1d). It is noticeable that the shifted value of LiDFOB-FDMA electrolyte is larger than that of LiDFOB-DME electrolyte, indicating the stronger electrostatic interaction of DFOB^−^ anion with H_FDMA_ as compared to that of DFOB^−^ anion with H_DME_. With the introduction of HTE diluent, the corresponding ^1^H NMR signals shift downfield. This phenomenon is ascribed to the strong electrostatic interaction between anion and HTE, which competes with its interaction with H_solvent_ and thus reduces the shield effect of the anion on the solvent. However, the larger downfield displacement shows that the more significant deshielding effect appears for DDH electrolyte as compared with that of DFH electrolyte, reflecting a stronger competitive electrostatic interaction of DFOB^−^ anion -H_HTE_ in DDH than that in DFH electrolyte.

Furthermore, ^1^H NMR of HTE further validates the presence of electrostatic interaction (Fig. 1e). Driven by the electron-withdrawing effect of ether-based oxygen in DME and carboxyl oxygen in FDMA, both the mixture of solvents and HTE exhibit the upfield shift (Fig. S3a) [24]. In contrast to the weak shift from 4.300 to 4.295 ppm is observed in FDMA/HTE mixture, the DME/HTE displays a larger shift value of 0.009 ppm, suggesting the greater interaction between DME and HTE, as also verified by the calculation (Fig. S3b–c). With the introduction of LiDFOB salt, the signal of H nuclei in HTE continues to shift downward due to the electrostatic effect between DFOB^−^ (with electron-donating property) and HTE. Notably, the shifted value of 0.006 ppm in DFH electrolyte is lower than that in DDH electrolyte (0.008 ppm), which implies the stronger interaction of DFOB^−^ anion with H_HTE_ than that with H_DME_ in the DDH electrolyte. While in DFH electrolyte, partial DFOB^−^ anions can interact with FDMA molecules, showing a minor decrease in downfield displacement because of the competitive interactions between H_FDMA_ and H_HTE_ toward anion. Overall, these results demonstrate the electrostatic interaction between anion and solvent/diluent, where the DFOB^−^ anion is apt to interact with H_HTE_ in the DDH electrolyte while interactions of the anion toward H_FDMA_ and H_HTE_ are almost equivalent in DFH electrolyte. The latter is beneficial for regulating the anions at the inner of solvation structure, as will be discussed later.

### Solvation Sheath Characterization of Electrolytes.

**Fig. 2:**
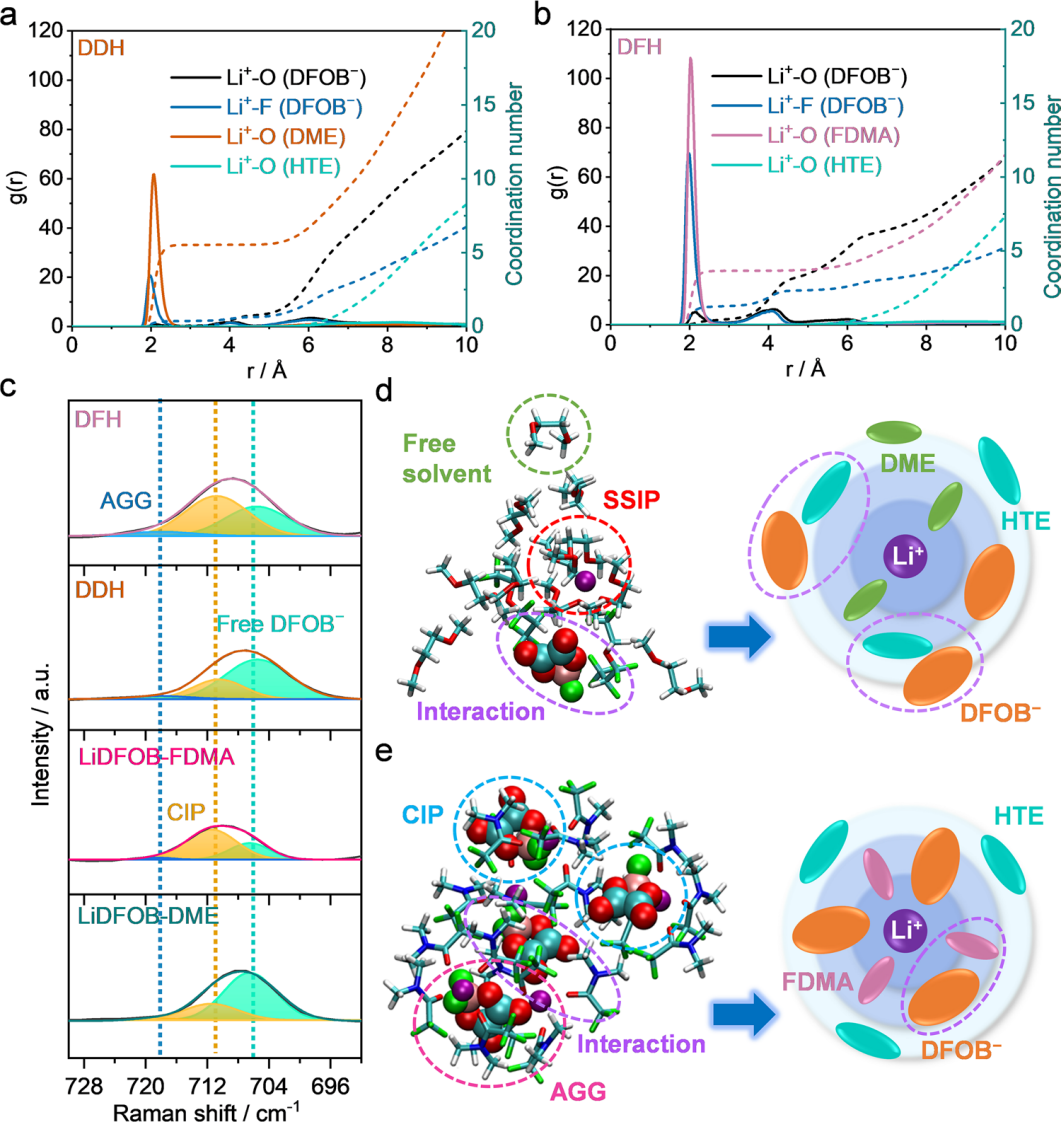
Coordination solvation chemistry for electrolytes. Li^+^ radial distribution function obtained from MD simulations of **a** DDH and **b** DFH electrolytes. Solid lines represent g(r) while dash lines represent coordination number. **c** Raman spectra of LiDFOB-DME, LiDFOB-FDMA, DDH, and DFH electrolytes. Li^+^-solvated structure in **d** DDH and **e** DFH electrolytes after simulation and the corresponding 2D schematic diagrams

Generally, solvation structure of commercial carbonate electrolytes mainly consists of free anion, solvent molecule, and solvated Li^+^ cation [5, 6]. Due to the unstable reduction products of solvents (*e.g.*, Li_2_O, ROCO_2_Li and ROLi), such an anion-free solvation is difficult to construct a robust interface for achieving long-term cycling of LMBs [6, 25]. To disclose the effect of electrostatic interaction on Li^+^ solvation sheath in the electrolyte, MD simulation and Raman characterization were carried out. RDF data of DDH electrolyte exhibits two typical peaks at ~ 2.1 Å around the centroid, which can be assigned to DME and DFOB^−^ species. Based on the peak intensities, the first Li solvation sheath is dominated by large amount of DME molecules and few DFOB^−^ anion, which indicates that the DDH electrolyte display a typical SSIP-rich solvation structure (Figs. 2a and S4a). Meanwhile, the coordination numbers of DME and DFOB^−^ are 2.766 and 0.64, respectively (Fig. 2a). In contrast, the RDF pattern of DFH electrolyte delivers three peaks at positions around 2.1 Å, which are attributed to Li^+^…O(FDMA), Li^+^…F(DFOB^−^), and Li^+^…O(DFOB^−^), respectively (Fig. 2b). The coordination numbers of FDMA and DFOB^−^ are 3.657 and 2.33, respectively, indicating that the solvation sheath is dominated by the characteristic contact-ion pair (CIP, one DFOB^−^ coordinating to one Li^+^), accompanied by some aggregations (AGG, one DFOB^−^ coordinating to two Li^+^ or more) in DFH electrolyte (Figs. 2b and S4b). Briefly, such an anion-rich solvation structure in the DFH electrolyte is completely different from the DME solvent molecule-dominated solvation in the DDH electrolyte. In addition, no peaks related to coordination between Li^+^ and HTE are observed in these two electrolytes, which suggests that HTE acts as a diluent in these electrolytes.

Raman vibrational spectroscopy of electrolytes and their components was performed to further investigate the Li^+^ solvation sheath of electrolytes. With the dissolution of LiDFOB into DME, the two peaks of free DME molecule at ~ 850 and 820 cm^−1^ decrease (Fig. S5a) [6, 26, 27]. Meanwhile, two extra bands appear at around 873 and 840 cm^−1^, corresponding to Li^+^-coordinated DME [6, 28]. Similarly, the signals of free FDMA molecules (about 756 cm^−1^) diminish when LiDFOB dissolves, which are replaced by a new band at ~ 762 cm^−1^ (Li^+^-coordinated FDMA) in FDMA-based electrolyte (Fig. S5b) [29]. After the introduction of HTE, the presence of Li^+^-coordinated solvent peaks indicates some solvent molecules remain in solvation sheath. Furthermore, the participation of anion in the solvation sheath was investigated (Fig. 2c). In LiDFOB-FDMA electrolyte, the CIP (at 711 cm^−1^) stays at the solvation shell, as along with some free DFOB^−^ anions (at 706 cm^−1^) [30], showing a definite weak-solvated ability of FDMA. While the situation is quite different in LiDFOB-DME electrolyte, where the majority of DFOB^−^anions are present in the states of free anions and few CIP due to the strong solvation ability of DME with Li^+^ [5]. With addition of HTE, no obvious change in intensity is observed for these peaks of free DFOB^−^ and CIP in DDH electrolyte. This result suggests that the strong electrostatic interaction between DFOB^−^ and HTE that stays outside interferes with the access of anion to the inner Li^+^ solvation sheath. By contrast, the peak intensities of CIP and AGG clusters (near 718 cm^−1^) enhance in DFH electrolyte [30], indicating that more anions exist in the solvation sheath, which are well consistent with MD calculations (Fig. 2b). Such effect is ascribed to the concomitant moderate electrostatic interaction between DFOB^−^ and HTE or FDMA. Overall, in DDH electrolyte, those DFOB^−^ anions stays outside of solvation sheath due to the strong electrostatic interaction between anion and HTE (Fig. 2d). Whereas, the FDMA can facilitate anion to get close to the inner layer, contributing to forming an anion-rich solvation sheath (Fig. 2e).

### Li Metal Morphology and Interfacial Chemistry

**Fig. 3:**
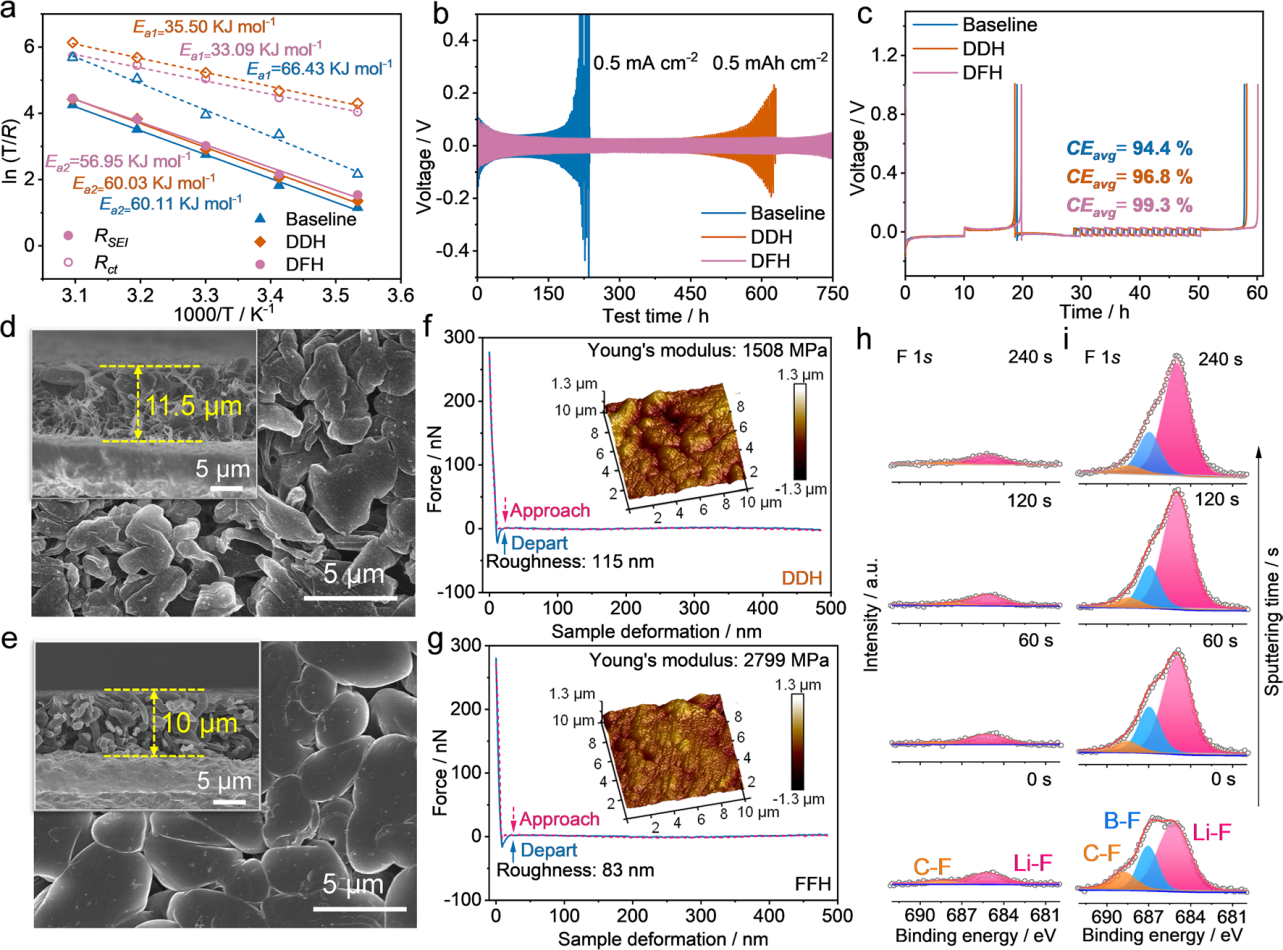
Li plating/stripping behavior and interfacial characterization. **a** The activation energies of *R*_SEI_ and *R*_ct_ derived from Nyquist plots of cycled Li||Li symmetric cells with baseline, DDH, and DFH electrolytes. **b** Voltage profiles of Li||Li symmetric cells using baseline, DDH, and DFH electrolytes at 0.5 mA cm^−2^ with a cutoff capacity of 0.5 mAh cm^−2^. **c** *CE*_avg_ tests of Li plating-stripping in Li||Cu cells using baseline, DDH, and DFH electrolytes at 0.5 mA cm^−2^ with capacity of 0.5 mAh cm^−2^. Top and cross-sectional (shown in inset) FE-SEM images of the Li deposition obtained by plating 1 mAh cm^−2^ Li on Cu substrate at 0.2 mA cm.^−2^ in Li||Cu cells using **d** DDH and **e** DFH electrolytes. Force–displacement plots of **f** DDH and **g** DFH electrolytes derived SEI. Corresponding three-dimensional atomic force microscope (3D-AFM) scanning images of SEI layers are shown in insets. F 1* s* in-depth XPS spectra of the Cu substrate obtained from the Li||Cu cells using **h** DDH and **i** DFH electrolytes

Such an anion-dominated solvation structure is potential to reduce the electrolyte conductivity due to the sparse free anions and cations, rendering a relatively low conductivity of 2.5 mS cm^−1^ for DFH electrolyte (Fig. S6) [31]. However, DFH electrolyte exhibits an Li-ion transfer number (*t*_*Li*_^+^) of 0.59, higher than that with other electrolytes (*t*_*Li*_^+^  = 0.40 for baseline and *t*_*Li*_^+^ = 0.51 for DDH electrolyte) (Fig. S7). This is attributed to the anion-rich solvation in the DFH electrolyte, which provides a facile de-solvation stage and a superior interface for ion diffusion [32]. The measurement of exchange current density further reveals the fast Li^+^ ion transfer kinetics in DFH electrolyte (Fig. S8). To further evaluate this, temperature-dependent EIS measurements of Li||Li cells were performed to obtain the activation energy of the Li deposition/stripping process (Figs. 3a and S9). The energy barriers for the diffusion of Li^+^ through the SEI layer and de-solvation of Li^+^ at the interface, which originated from the SEI (*R*_SEI_) and ion transfer resistance (*R*_ct_), are denoted as *E*_a1_ and *E*_a2_, respectively [33]. The *E*_a1_ for DFH electrolyte (33.09 kJ mol^−1^) is lower than that for the baseline electrolyte (66.43 kJ mol^−1^) and the DDH electrolyte (35.50 kJ mol^−1^), indicating that the anion-derived SEI layer formed in DFH electrolyte can facilitate a fast Li^+^ transport kinetics. Meanwhile, *E*_a2_ value of baseline, DDH, and DFH electrolytes are 60.11, 60.03, and 56.95 kJ mol^−1^, respectively. The reduced de-solvation energy barrier implies that the amide solvent and anions in the Li^+^ solvation sheath synergistically promote the fast dissociation of solvated Li^+^.

To investigate the stability of Li metal anodes in different electrolytes, the voltage evolution of symmetric Li||Li cells at 0.5 mA cm^−2^ was recorded. As shown in Fig. 3b, the symmetric Li||Li cell using conventional carbonates present the dramatically increased overpotential with the cycling time due to the continuous thickening of non-uniform SEI layer [34], which exceeds 500 mV at 230 h. Without HTE addition, LiDFOB-DME and LiDFOB-FDMA electrolytes can only maintain the cycling time of 150 and 430 h for Li||Li symmetric cells, respectively (Fig. S10). For DDH electrolyte, the overpotential of Li||Li cells remains stable until 450 h, then increases significantly, and finally fails with a dendrite-induced short circuit at 650 h. In contrast, the DFH electrolyte endows the symmetric cell with a stable voltage hysteresis of 50 mV throughout a 750-h cycling. The voltage variation of symmetric Li||Li cells under different current densities also confirm the superiority of DFH electrolyte (Fig. S11). The reversibility of Li plating/stripping in various electrolytes can be further measured in Li||Cu cells, as evaluated by the average Coulombic efficiency (*CE*_avg_) **(**Fig. 3c). The Li||Cu cells using DFH electrolyte exhibit a high *CE*_avg_ of 99.3%, much higher than those of reference electrolytes (94.4% for baseline electrolyte, and 96.8% for DDH electrolyte). Furthermore, the Li plating morphologies on Cu substrates in various electrolytes are characterized by field emission scanning electron microscope (FE-SEM). The plating Li with 2 mAh cm^−2^ capacity in the baseline electrolyte shows a highly loose and needle-like dendrites, which induces a continuous electrolyte depletion and a thickening of Li layer (around 14.8 μm, Fig. S12). For DDH electrolyte, relatively dense bulk Li deposition and a few dendrites are observed with a thickness of 11.5 μm (Fig. 3d). In comparison, when DFH electrolyte is adopted, the deposited Li exhibits a compact morphology with aggregated monoliths (Fig. 3e). Moreover, the thickness of Li deposition is confined to 10 μm (the inset of Fig. 3e), which is very close to the theoretical value (9.7 μm). Such a round-shaped and uniform Li deposition effectively minimizes the surface area to control the parasitic reactions at the Li/electrolyte interface, achieving a reversible Li plating/stripping behavior.

Atomic force microscopy (AFM) and in-depth X-ray photoelectron spectroscopy (XPS) measurements were performed to analyze the details of SEI layer in various electrolytes. As shown in Figs. S13 and S14a, the SEI layer formed in the baseline electrolyte provides a Young's modulus as low as 398 MPa, and its surface roughness is as high as 214 nm. The SEI compositions are identified as some organic compounds (*e.g.*, ROCO_2_Li) stemming from the decomposition of carbonate solvents and inorganics (*e.g.*, Li_x_PO_y_F_z_) from the reduction products of LiPF_6_ salt (Figs. S15a, S16a and S17a) [25, 35]. For DDH electrolyte, the mechanical strength of SEI layer is improved to 1,508 MPa (Fig. 3e), which was attributed to the decreased organics (Fig. S12b) and the presence of Li_2_O (529 eV in O 1* s* spectrum, Fig. S16b) and slight LiF (684.0 eV in F 1* s* spectrum, Fig. 3g) [36]. Meanwhile, the roughness is reduced to 115 nm due to the denser Li deposition (Fig. S14b). Owing to the presence of an anion-rich solvation, the as-formed SEI layer in DFH electrolyte contains more inorganic species with high Young's modulus. As shown in Figs. 3f and S14c, a high Young's modulus of 2,799 MPa as well as a controlled roughness (~ 83 nm) were achieved with DFH electrolyte. The large mechanical strength is ascribed to the formation of a large amount of LiF and B-containing species (*i.e.*, B-F at 687.0 eV in F 1* s* and 193.5 eV in B 1* s* spectrum, and B-O at 532.5 eV in O 1* s* and 192.0 eV in B 1* s* spectrum, Figs. 3h, S16c and S17c) as the decomposition product of LiDFOB salt [37]. It is widely recognized that LiF possesses high mechanical strength and exhibits high interfacial energy toward Li anodes, remarkably improving the stability of SEI layer to suppress the growth of dendritic Li [38–40]. Moreover, the amount of LiF species deliver a gradient increase from outer to inner layer of the SEI. Notably, the great content difference of B-containing species reveals the significance of anion-rich solvation sheath from the regulation of electrostatic interaction (Fig. S17b–c). To validate the contribution of anion-rich solvation sheath to SEI chemistry, in-depth XPS detection was also conducted by using both LiDFOB-DME and LiDFOB-FDMA as the reference electrolytes (Figs. S18 and S19). Briefly, in DFH electrolyte, the tailored DFOB^−^-rich Li^+^ solvation structure facilitates the construction of a gradient-heterostructure SEI layer characterized by an inner layer rich in inorganic LiF and B species and an outer layer composed of organic components.

### Electrochemical Performances Evaluation

**Fig. 4:**
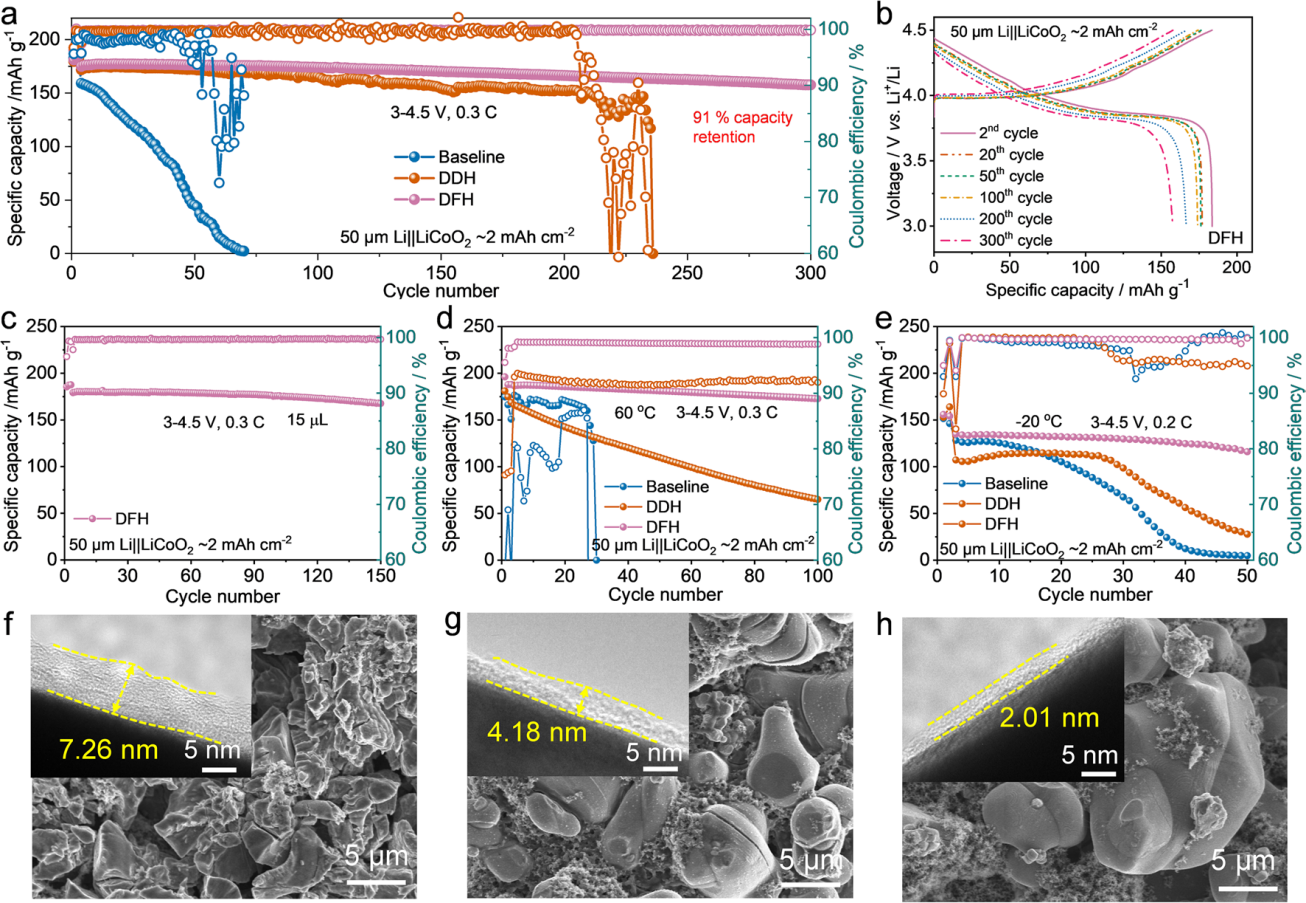
Cyclic performances of Li||LiCoO_2_ full batteries and LiCoO_2_ cathode morphology after cycling. **a** Long-term cycling performance of Li||LiCoO_2_ cells using baseline, DDH, and DFH electrolytes. **b** Charge–discharge voltage profiles of a Li||LiCoO_2_ cells employing DFH electrolyte at different cycles. **c** Cycling performance of Li||LiCoO_2_ cells using DFH with 15 μL electrolyte. Cycling performance of Li||LiCoO_2_ cells using baseline, DDH, and DFH electrolytes at **d** 60 °C and **e** − 20 °C. FE-SEM and TEM images of LiCoO_2_ cathode cycled in **f** baseline, **g** DDH, and **h** DFH electrolytes for 50 cycles at 0.3C

Figure 4a shows the long-term cycling performances of high-voltage LMBs in various electrolytes with the high-loading LiCoO_2_ cathode (11 mg cm^−2^, about 2 mAh cm^−2^) and the thin Li foil (50 μm). Within the voltage range of 3–4.5 V, the Li||LiCoO_2_ cell using baseline electrolyte display a rapid capacity decay and a fluctuating CE (Figs. 4a and S20a), which fails after 70 cycles (capacity decreases to zero). The Li||LiCoO_2_ cells with LiDFOB-DME electrolyte also show a sharp capacity fading within 125 cycles (Fig. S21). Benefiting from the efficacy of HTE diluent, the cycling performance of the cells using DDH electrolyte is improved to 205 cycles. However, the dramatic oscillation of capacity is observed during the subsequent 30 cycles. The *CE*_avg_ of the full cell is ~ 99.4% over 205 cycles, which also fluctuates abnormally with values between 60 and 90% for the subsequent cycles (Figs. 4a and S20b). For comparison, the cells using LiDFOB-FDMA electrolyte can display a long-term cycling for 300 times but a low capacity retention of 49% (Fig. S21). Impressively, DFH electrolyte enables the Li||LiCoO_2_ cell to cycle stably over 300 cycles at 0.3C rate, with a high-capacity retention of 91% and a steady *CE*_avg_ of up to 99.8%. Furthermore, the typical charge–discharge curves show a minimum voltage drop and overpotential within 300 cycles (Fig. 4b), which is in sharp contrast to the overpotential growth in baseline and DDH electrolytes (Fig. S20b). In addition, DFH electrolyte enhances the cycling stability of LMBs when at a high current density of 1C (76% capacity retention within 300 cycles, Fig. S22a), under the ultrahigh voltage of 4.6 V (74% capacity retention after 150 cycles, Fig. S22b), and even in the Li-free Cu||LiCoO_2_ cells (63% capacity retention for 50 cycles) (Fig. S22c). When the electrolyte amount is reduced from the basic 60 μL to the rigid 15 μL, Li||LiCoO_2_ cells still maintain a capacity of 167.6 mAh g^−1^ at the 150^th^ cycle (~ 94% of the initial capacity) with stable *CE*_avg_ as high as 99.7% (Fig. 4c).

Moreover, DFH electrolyte exhibits a wide-range temperature feature for meeting the application of LMBs under the different scenarios. Under a high-temperature of 60 °C (Fig. 4d), Li||LiCoO_2_ cells with the baseline electrolyte display a fluctuated capacity decline and fail with a capacity of 0 at the 30^th^ cycle. For DDH electrolyte, a fast capacity drop from 174.8 to 66 mAh g^−1^ within 100 cycles is observed, together with a low *CE*_avg_ of 91%. These poor performances are attributed to the formation of the thermally unstable electrode/electrolyte interfaces, leading to the electrolyte depletion and the failure of cathode materials structure [41, 42]. Impressively, the cycle stability is effectively enhanced in DFH electrolyte, as evidenced by the high average CE of 99.3% and a capacity retention of 92% after 100 cycles. When the operating temperature is decreased to − 20 °C, Li||LiCoO_2_ cells can deliver an initial capacity of 135.0 mAh g^−1^ with 86% retention at 50^th^ cycle, indicating the fast Li^+^ transport kinetics through interfaces and low energy barrier of ion diffusion in DFH electrolyte. In comparison, the cells using both baseline and DDH electrolytes present a fast capacity decline with fluctuating CE due to the decreased ionic conductivity and the sluggish interfacial charge transfer kinetics [43]. Benefiting from the superior Li^+^ diffusion with DFH electrolyte, Li||LiCoO_2_ cells also exhibit an improved rate performance (Fig. S23). Furthermore, the comparatively smaller interfacial resistances (*R*_sei_ and *R*_ct_) and the corresponding resistance evolution for Li||LiCoO_2_ cells using DFH electrolyte suggests the formation of a thin and robust electrolyte/electrode interface with low resistance (Fig. S24).

As a post-mortem procedure, TEM and XPS analysis of LiCoO_2_ cathode after cycling at 4.5 V *vs*. Li/Li^+^ in different electrolytes were conducted. Compared with the pristine LiCoO_2_ electrode (Fig. S25), the LiCoO_2_ cathode cycled in the conventional carbonates presents an apparent particle-crushing with an uneven cathode electrolyte interphase (CEI) (~ 7.26 nm, Fig. 4f). In addition, the CEI compositions are identified as large amounts of organic compounds and minor LiF from electrolyte oxidative decomposition [44], meanwhile, the presence of metal oxide (M–O) bonds implies that the highly active LiCoO_2_ cathode cannot be effectively passivated (Figs. S26a, S27a, and S28a) [37]. For DDH electrolyte, some cracks in bulk particles appear on LiCoO_2_ cathode, which is covered by LiF-rich CEI film with a thickness of 4.18 nm (Figs. 4g and S28b). By contrast, the LiCoO_2_ cathode obtained from DFH electrolyte keeps intact (Fig. 4h). Meanwhile, a compact and thinner CEI film (about 2.01 nm) is formed on the LiCoO_2_ surface, which is enriched with LiF, C-F and N-containing species from FDMA degradation (inset of Figs. 4h, S28c, and S29) [45]. This heterogeneous CEI film is conducive to Li^+^ transportation and favorable for uniform ion distribution [46]. These results demonstrate that such an electrolyte with anion-rich solvation regulated by electrostatic interaction can not only facilitates the formation of a robust SEI layer for the reversible Li plating/stripping, but also endows high-voltage LiCoO_2_ cathode with superior electrochemical performances without electrolyte decomposition and structural destruction.

## Conclusions

In summary, a novel mechanism of electrostatic interaction between anion and solvent on regulating solvation sheath is proposed. As validated by theoretical simulation and experimental investigation, the moderate interaction between the DFOB^−^ anion and the FDMA solvent contributes to the participation of abundant anions in the solvation sheath. The anion-derived SEI layer with gradient heterogeneous structure exhibits both superior mechanical strength and high stability, enabling a high Coulombic efficiency of ~ 99.3% in Li||Cu cells and a stable Li^+^ plating/stripping throughout 750-h prolonged cycling in symmetric Li||Li cells. Furthermore, benefiting from the fast ion transport kinetics through CEI film, excellent electrochemical performances for Li|LiCoO_2_ full cell are achieved under a high charge voltage of 4.5 V (even up to 4.6 V), the depleted electrolyte of 15 μL, and a wide temperature range from − 20 to 60 °C. This work discloses the effect of the relationship between anion and solvent on solvation structure regulation and opens up a new direction for electrolyte design of LMBs.

## Supplementary Information

Below is the link to the electronic supplementary material.

Supplementary file1 (PDF 2522 kb)
