# Supplementary material for: Electrostatic Interaction Tailored Anion-Rich Solvation Sheath Stabilizing High-Voltage Lithium Metal Batteries
Source: Nanomicro Lett. 2022 Jul 21;14:147. doi: 10.1007/s40820-022-00896-4 (PMC9304530; doi:10.1007/s40820-022-00896-4)
Supplement: Supplementary file 1 — Supplementary file1 (PDF 2522 kb) [file 40820_2022_896_MOESM1_ESM.pdf]

Supporting Information for

# Electrostatic Interaction Tailored Anion-Rich Solvation Sheath Stabilizing High-Voltage Lithium Metal Batteries

Junru Wu[1, 2], Ziyao Gao[1, 2], Yao Wang[1], Xu Yang[1], Qi, Liu[1, 3], Dong Zhou[1], Xianshu Wang[1, *], Feiyu Kang[1, 2], Baohua Li[1, *]

[1] Tsinghua Shenzhen International Graduate School, Tsinghua University, Shenzhen 518055, P. R. China

[2] School of Materials Science and Engineering, Tsinghua University, Beijing 100084, P. R. China

[3] College of Materials Science and Engineering, Hunan University, Changsha 410082, P. R. China

*Corresponding authors. E-mail: xswang2016@m.scnu.edu.cn (Xianshu Wang); libh@sz.tsinghua.edu.cn (Baohua Li)

## Supplementary Figures and Tables

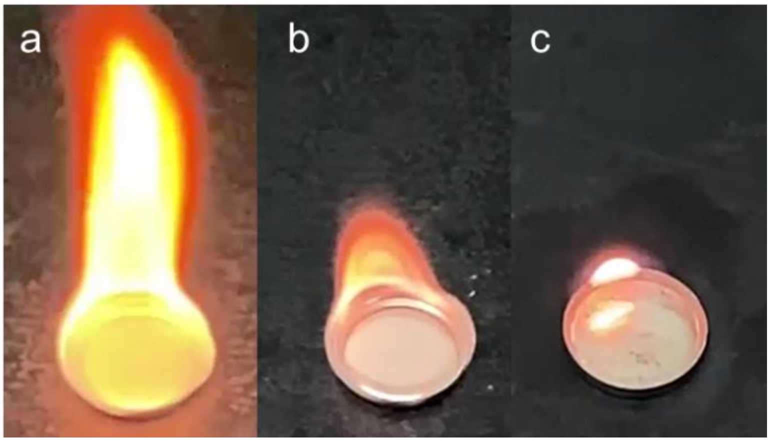


**Fig. S1** Combustion tests of **a** baseline, **b** DDH, and **c** DFH electrolytes

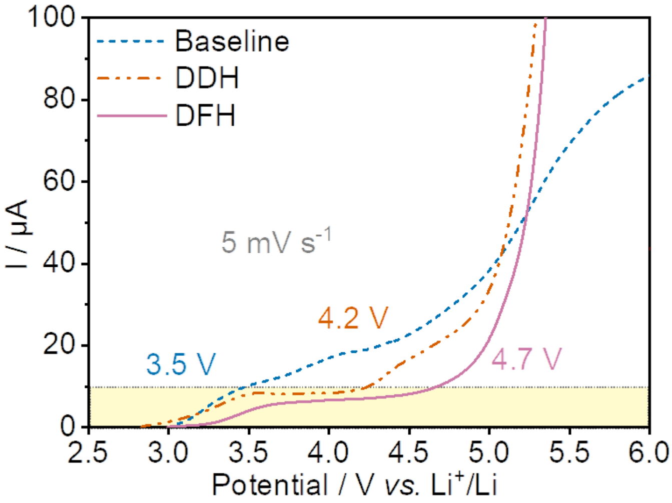


**Fig. S2** LSV curves of baseline, DDH, and DFH electrolytes at a scan rate of 5 mV $s^{-1}$, using stainless steel as the working electrode and Li foil as the counter and reference electrodes

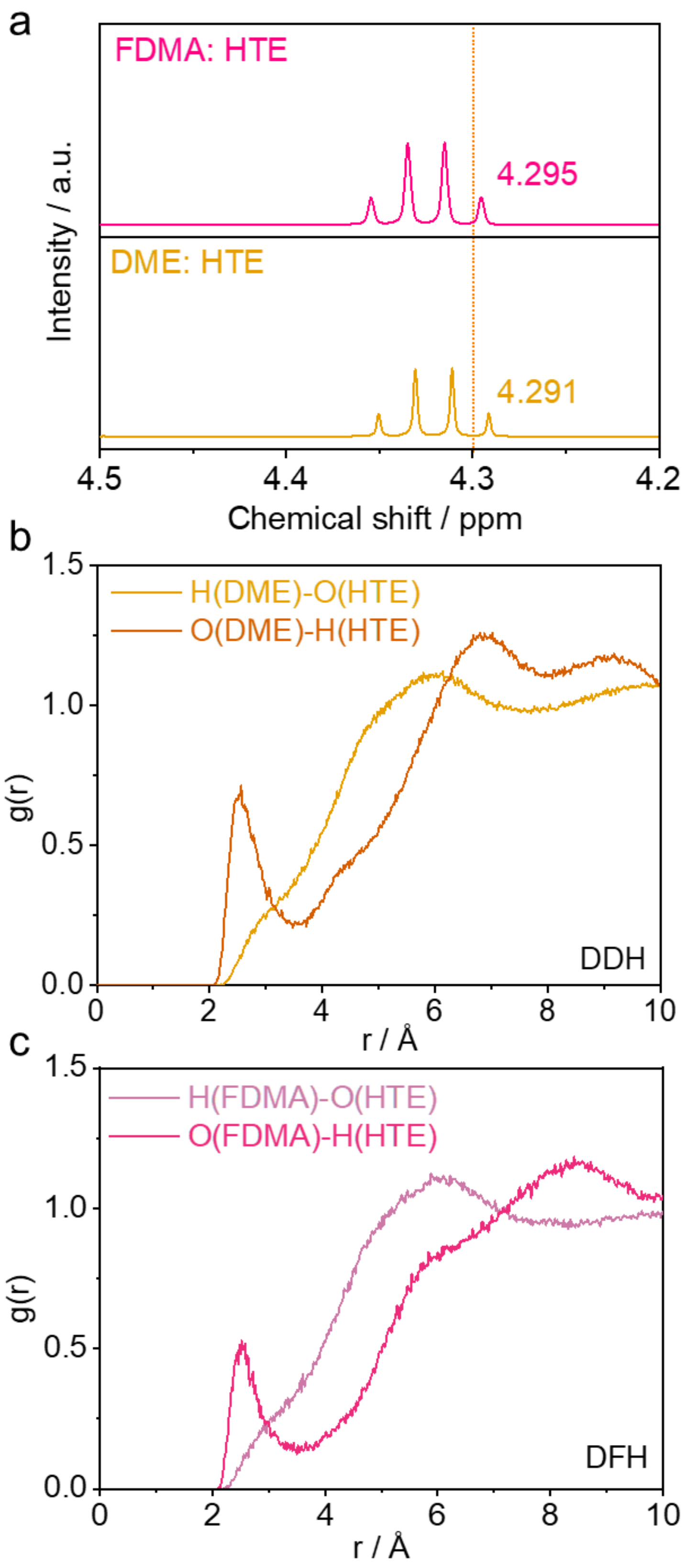


**Fig. S3 a** $^{1}H$ NMR spectra of pure HTE solvent, DME: HTE mixture, FDMA: HTE mixture. Radial distribution function between **b** DME and HTE solvent molecule in DDH and **c** FDMA and HTE solvent molecule in DFH electrolytes

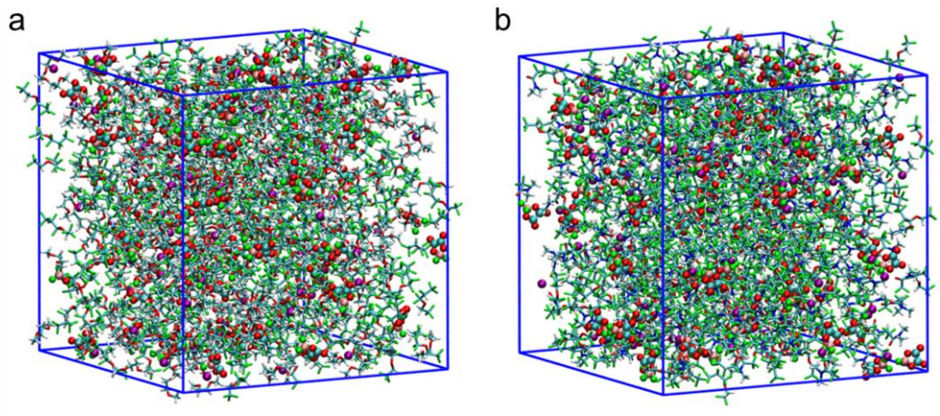


**Fig. S4** Snapshots obtained from MD simulations of **a** DDH and **b** DFH electrolytes

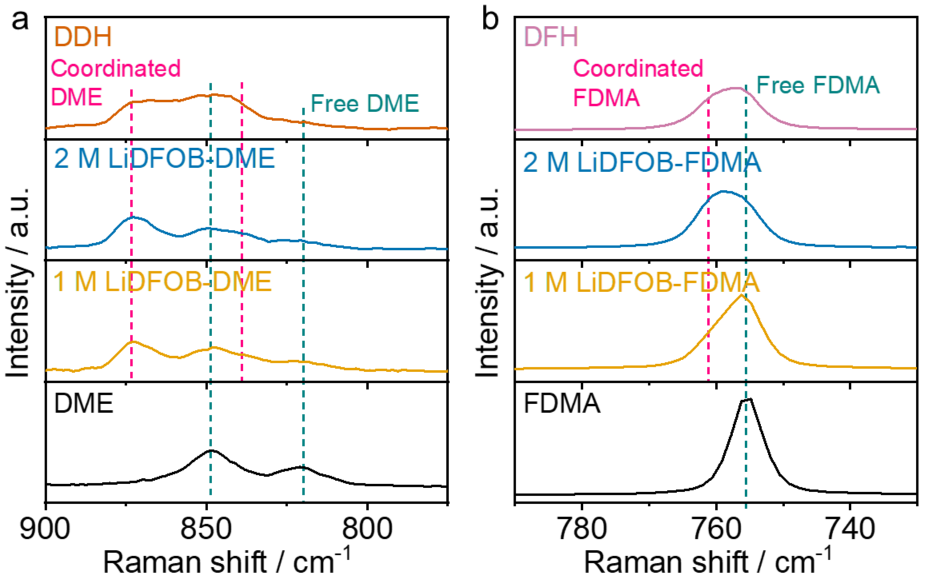


**Fig. S5** Raman spectra of **a** pure DME solvent, 1 M DFOB-DME, 2 M DFOB-DME, and DDH electrolytes and **b** pure FDMA solvent, 1 M DFOB-FDMA, 2 M DFOB-FDMA, and DFH electrolytes

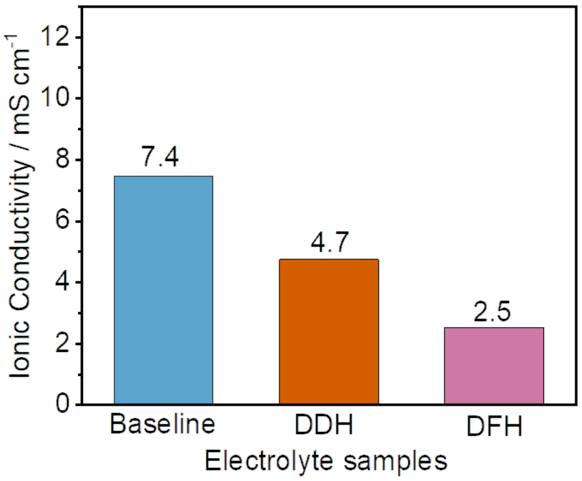


**Fig. S6** Ionic conductivities for baseline, DDH and DFH electrolytes at room temperature

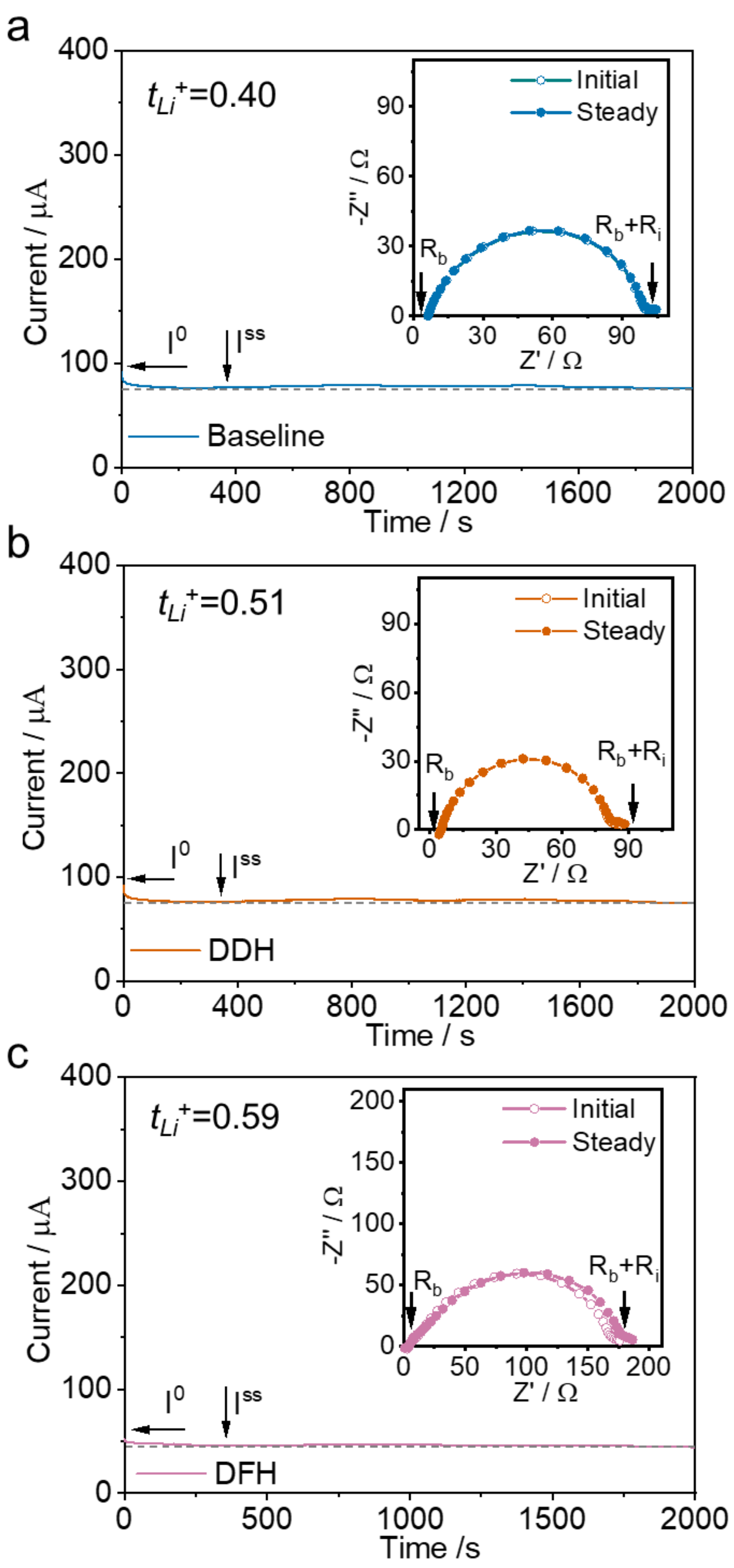


**Fig. S7** The chronoamperometry profile of the symmetric Li||Li cells using **a** baseline, **b** DDH, and **c** DFH electrolytes. The applied polarization voltage is 10 mV. The EIS spectra before and after the polarization are shown in inset

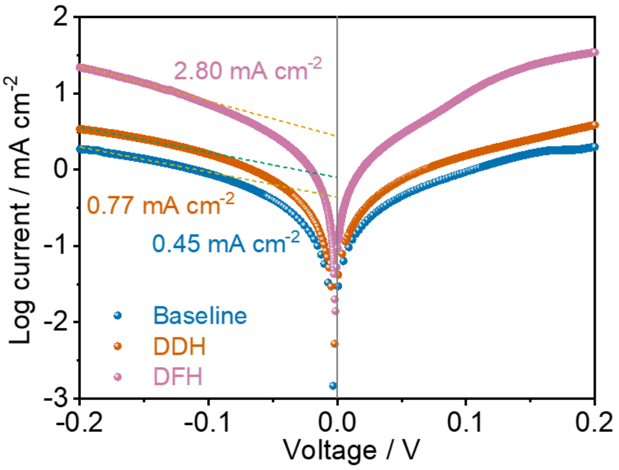


**Fig. S8** Tafel plots of Li plating/stripping in various electrolytes. Tafel plots are derived by plotting the overpotential of galvanostatic Li plating/stripping versus the natural log of the current density

By fitting, the exchange current density indicating ion transfer kinetics at the interface was obtained. The exchange current density (2.8 mA cm$^{-2}$) of DFH is over six times and three times larger than that of baseline (0.45 mA cm$^{-2}$) and DDH (0.77 mA cm$^{-2}$) electrolytes, respectively, demonstrating the fast $Li^+$ ion transfer kinetics.

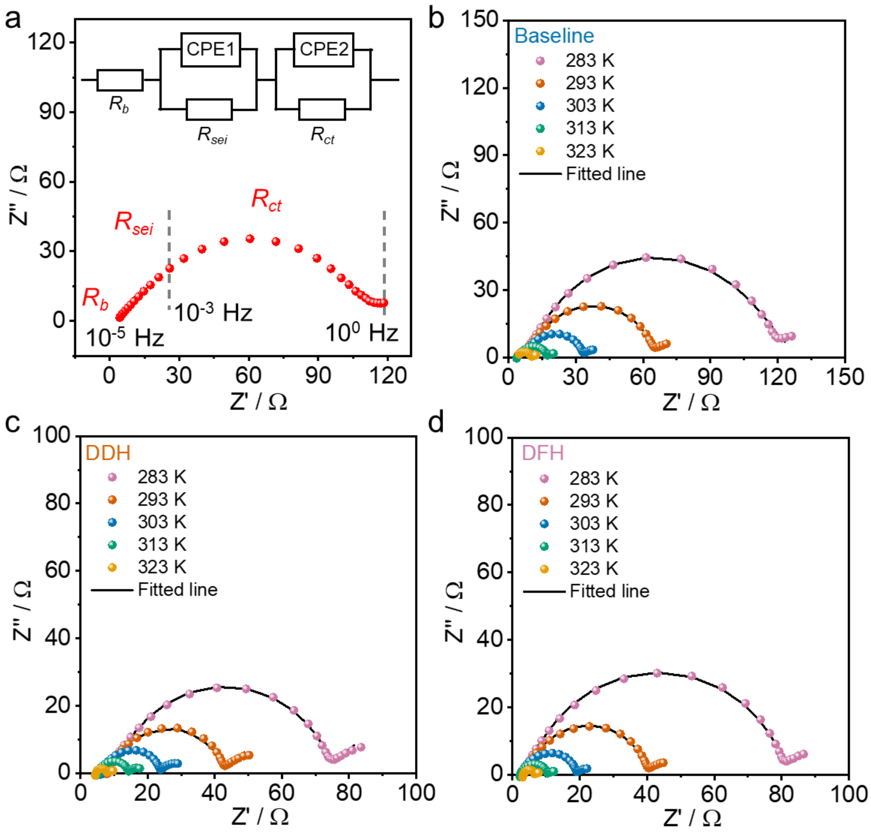


**Fig. S9 a** The equivalent circuit model and the corresponding Nyquist plot of Li||Li symmetric cells. $R_b$ represents the bulk resistance, reflecting the resistance of electrodes, electrolyte, and separator. The semi-circle of $R_{SEI}$ in the high-middle frequency range represents the resistance of $Li^+$ transport through the SEI, while the semi-circle of $R_{ct}$ in the low frequency range represents the de-solvation resistance of $Li^+$ before it enters the SEI. Nyquist plots for Li||Li symmetric cells using **b** baseline, **c** DDH, and **d** DFH electrolytes

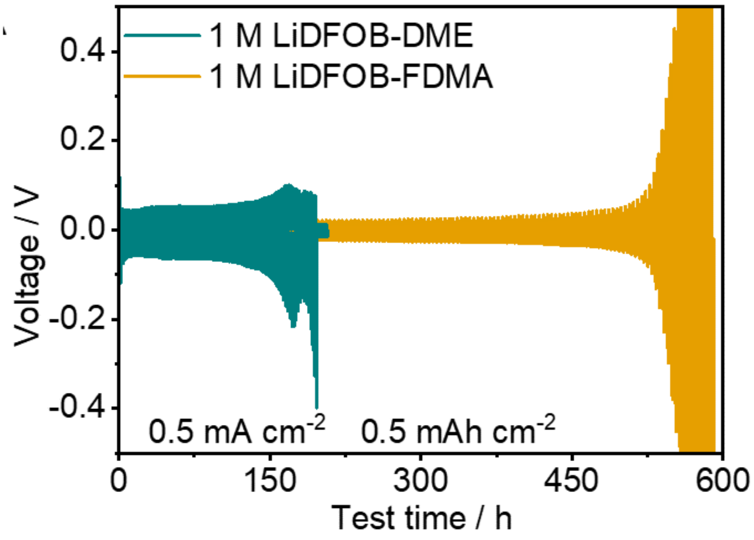


**Fig. S10** Voltage-time profiles of Li||Li using LiDFOB-DME and LiDFOB-FDMA electrolytes at a constant current density of 0.5 mA cm$^{-2}$ with a cut-off capacity of 0.5 mAh cm$^{-2}$

The Li||Li symmetric cells using LiDFOB-DME exhibit an increased overpotential significantly after 150 h and fail at 210 h, which is attributed to the formation of unstable SEI layer from the typical SSIP solvation structure (Figs. 2c and S5a). While LiDFOB-FDMA electrolyte can endow the cell with a slightly better cycling over 430 h, profiting from the definite CIP in its solvation (Fig. 2c).

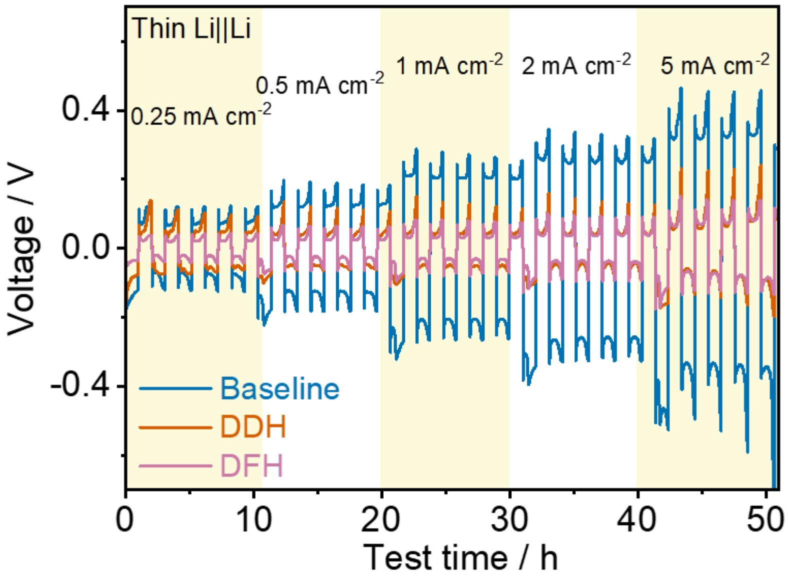


**Fig. S11**Voltage profiles of symmetric Li||Li cells at different current densities

As shown in Fig. S11, the voltage hysteresis of Li||Li cells with DFH electrolyte is 23, 24, 32, 41, and 70 mV at a current density of 0.25, 0.5, 1, 2, and 5 mA cm$^{-2}$, respectively. These lower values than that of baseline and DDH electrolytes, suggesting that DFH electrolyte endows the cells with a better current adaptivity.

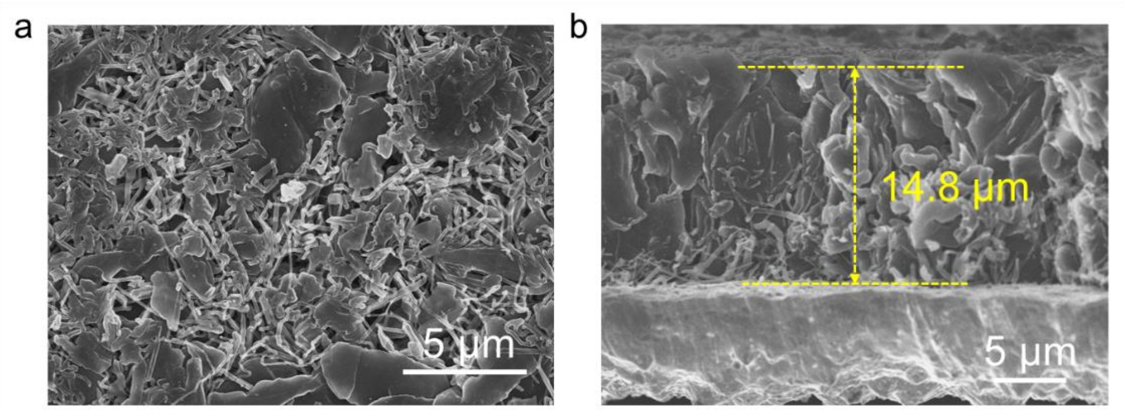


**Fig. S12 a** Top and **b** cross-sectional FE-SEM images of the Li deposition obtained by plating 1 mAh cm$^{-2}$ Li on Cu substrate at 0.2 mA cm$^{-2}$ in Li||Cu cells using baseline electrolyte

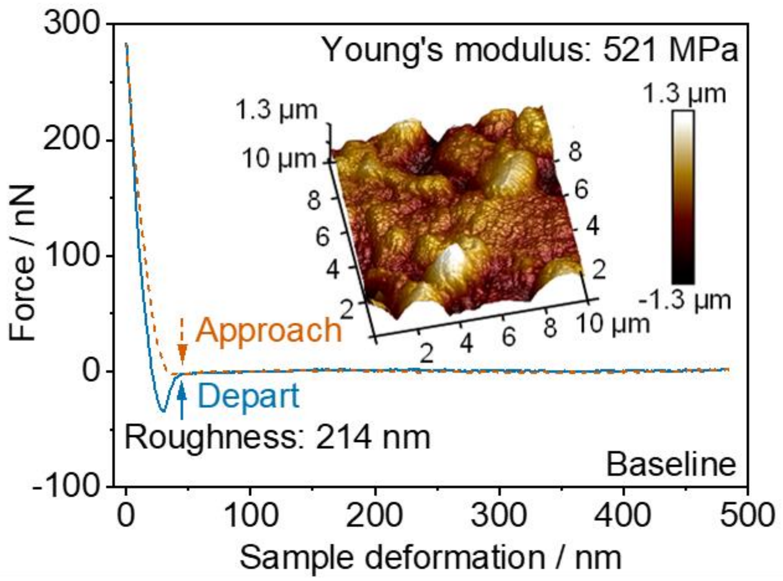


**Fig. S13** Force-displacement plots of baseline electrolyte derived SEI. Corresponding three-dimensional atomic force microscope (3D-AFM) scanning images of SEI layers are shown in inset

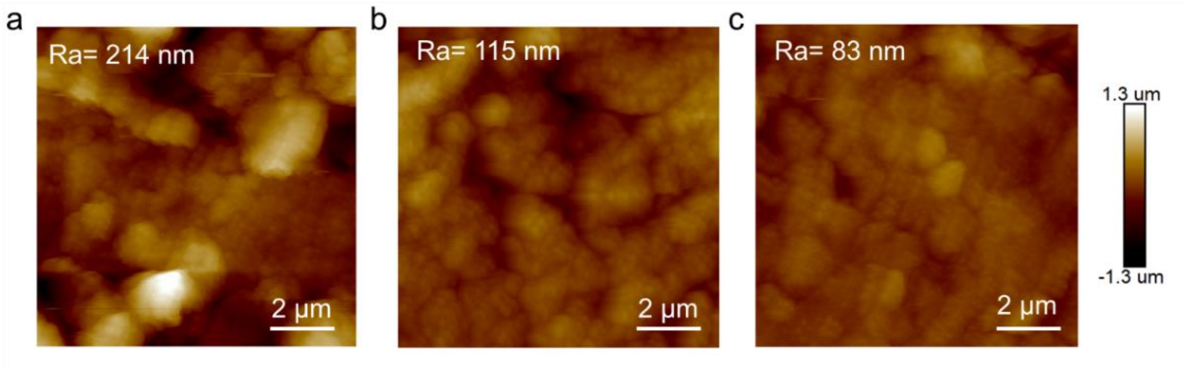


**Fig. S14** Two-dimensional (2D) AFM images of **a** baseline, **b** DDH, and **c** DFH electrolytes

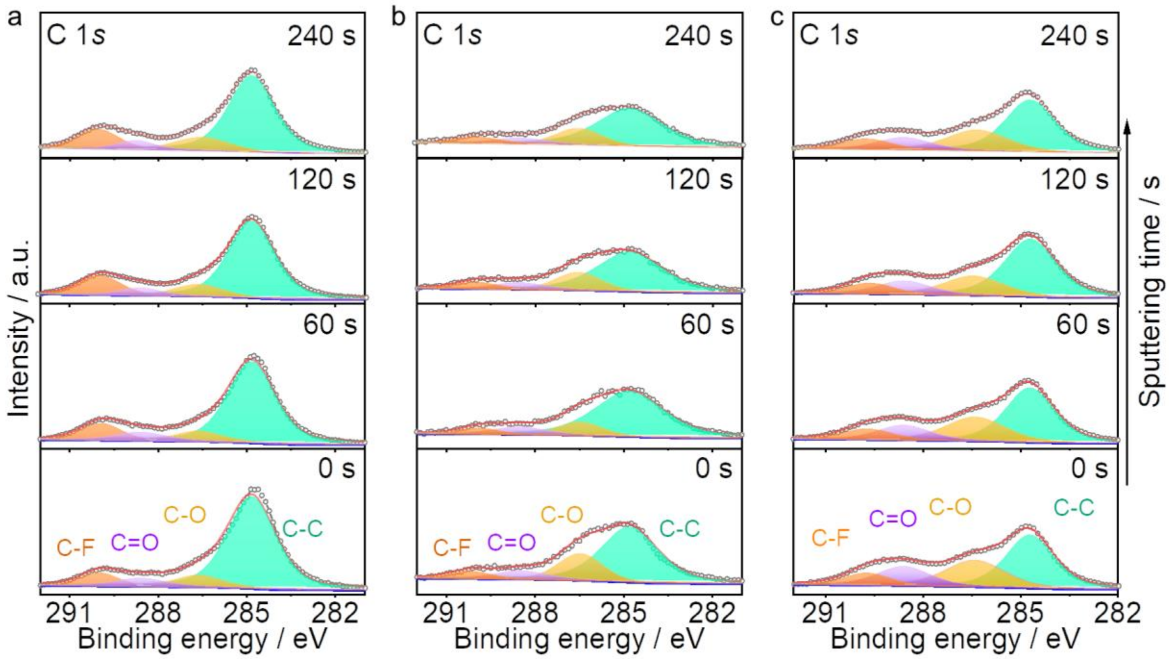


**Fig. S15** C 1*s* in-depth XPS spectra of the Cu substrate obtained from the Li||Cu cells using **a** baseline, **b** DDH, and **c** DFH electrolytes. C-C: 284.8 eV, C-O: 286.5 eV; C=O:288 eV; C-F: 290 eV [S1]

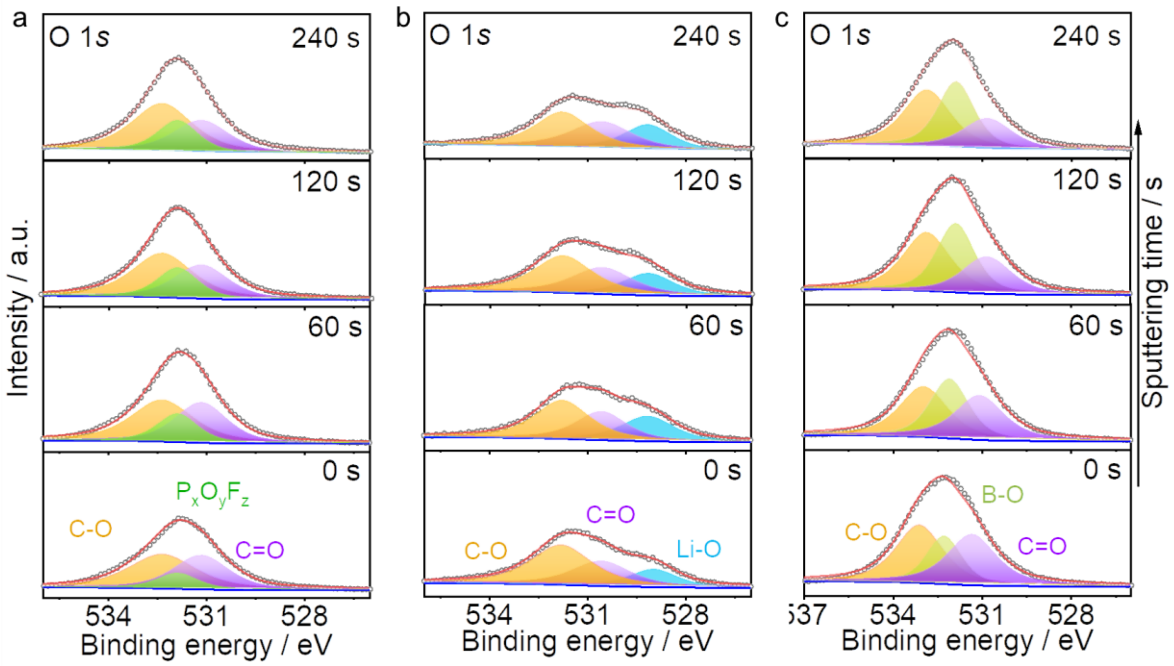


**Fig. S16** O 1*s* in-depth XPS spectra of the Cu substrate obtained from the Li||Cu cells using **a** baseline, **b** DDH, and **c** DFH electrolytes. $Li_2O$: 529 eV[S2]; C=O: 531.5 eV [S2]; C-O=533 eV [S3]; $P_xO_yF_z$: 532.0 eV [S4]; B-O: 532.5 eV [S5]

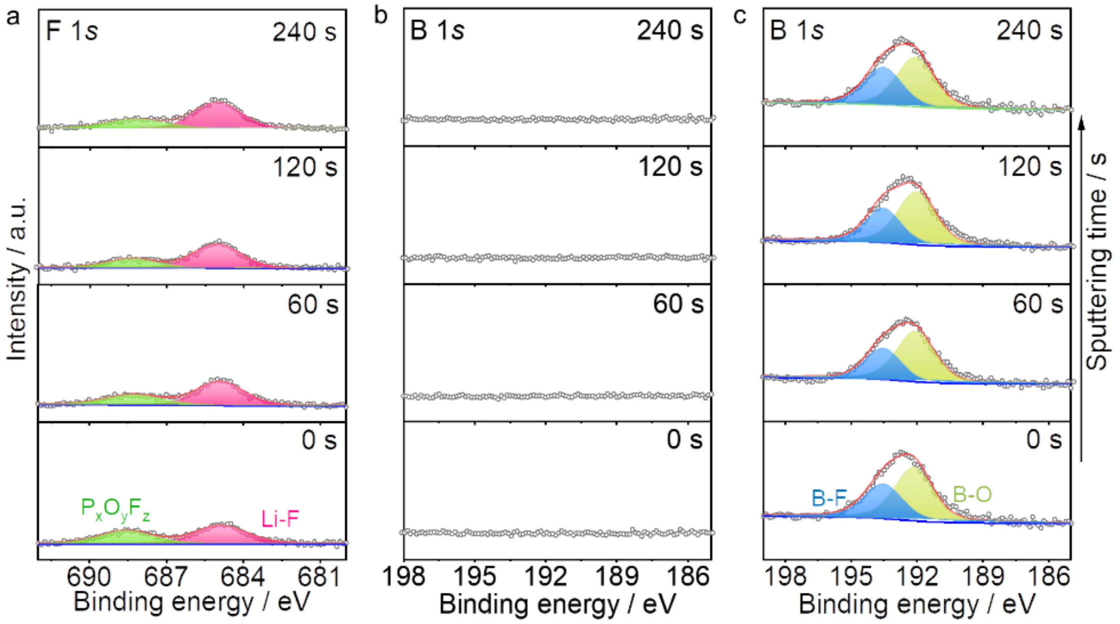


**Fig. S17 a** F 1*s* in-depth XPS spectra of the Cu substrate obtained from the Li||Cu cells using baseline electrolyte. B 1*s* in-depth XPS spectra of the Cu substrate obtained from the Li||Cu cells using **b** DDH and **c** DFH electrolytes. F 1*s*: LiF: 685 eV; $P_xO_yF_z$: 687 eV [S6]. B 1*s*: B-O:192 eV; B-F: 193.5 eV [S5]

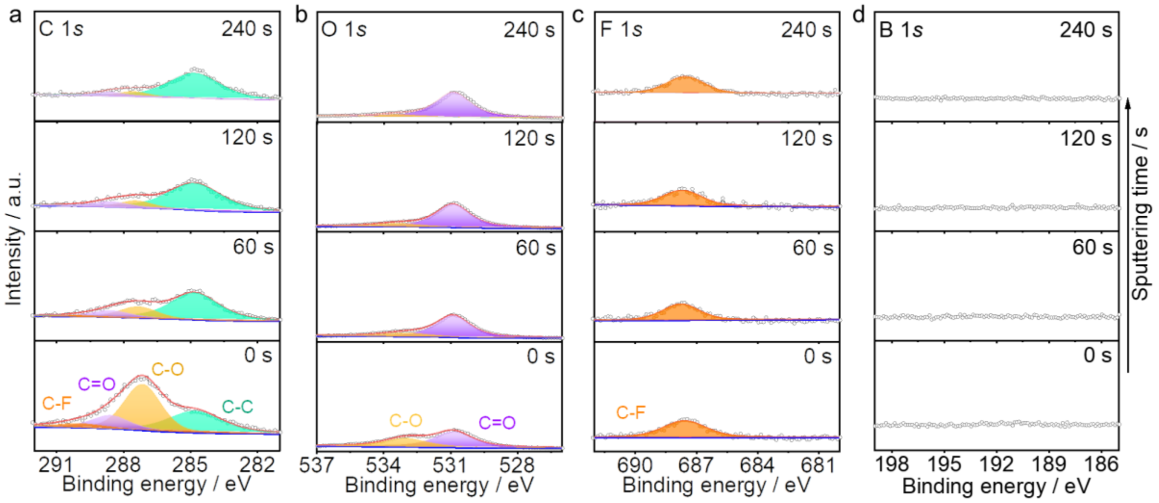


**Fig. S18 a** C 1*s*, **b** O 1*s*, **c** F 1*s*, and **d** B 1*s* in-depth XPS spectra of the Cu substrate obtained from the Li||Cu cells using LiDFOB-DME electrolyte. C 1*s*:C-C: 284.8 eV, C-O: 286.5 eV; C=O:288 eV; C-F: 290 eV [S1]. O 1*s*: C=O: 531.5 eV [S2]; C-O=533 eV [S3]; $P_xO_yF_z$: 532.0 eV [S4]; B-O: 532.5 eV [S5]. F 1*s*: C-F: 688 eV [S6]

As shown in Fig. S18, in LiDFOB-DME electrolyte, the formed SEI layer is mainly composed of organic component (e.g., $ROCO_2Li$, 284.8 and 287.0 eV in the C 1*s* and 531.0 and 533.5 eV in the O 1*s* spectrum) derived from decomposition of solvent and a few C-F (688.0 eV in the F 1*s* spectrum) from the reduction of $DFOB^-$ anions. The former is attributed to large amount of coordinated DME in the $Li^+$ solvation sheath, while the latter derives from the few CIP. Such organics-rich SEI cannot stabilize Li metal anode, resulting in the formation of Li dendrites and the continuous depletion of the electrolyte, and consequently deteriorating battery performance (Figs. S10 and S21).

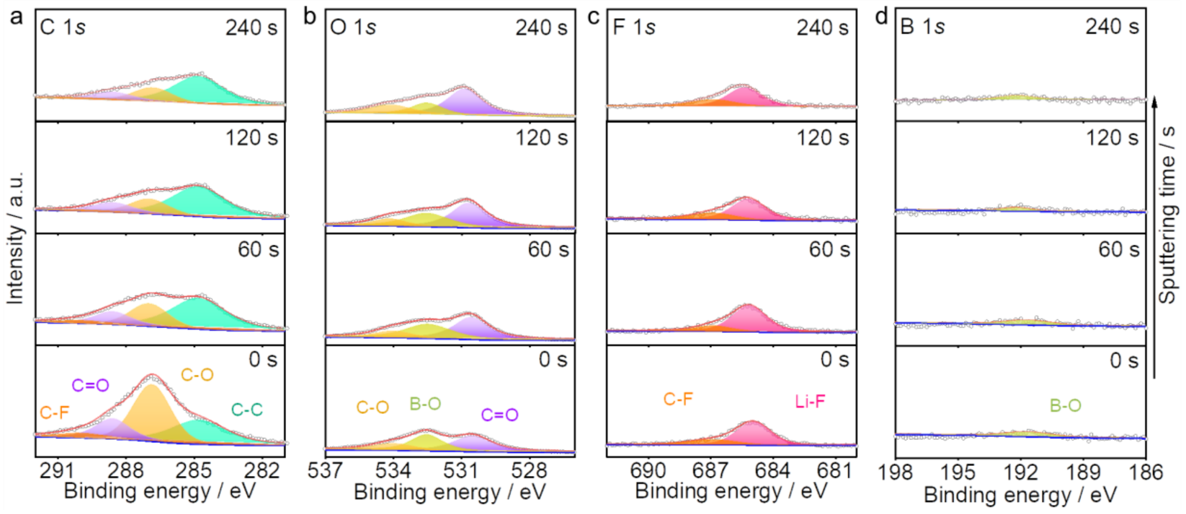


**Fig. S19** **a** C 1*s*, **b** O 1*s*, **c** F 1*s*, and **d** B 1*s* in-depth XPS spectra of the Cu substrate obtained from the Li||Cu cells using LiDFOB-FDMA electrolyte. C 1*s*:C-C: 284.8 eV, C-O: 286.5 eV; C=O:288 eV; C-F: 290 eV [S1]. O 1*s*: C=O: 531.5 eV [S2]; C-O=533 eV [S3]; $P_xO_yF_z$: 532.0 eV [S4]; B-O: 532.5 eV [S5]. F 1*s*: LiF: 685 eV; C-F: 688 eV [S6]. B 1*s*: B-O:192 eV [S5]

For LiDFOB-FDMA electrolyte, the SEI layer contains LiF (685.0 eV in F 1*s* spectrum) and trace amounts of B-O species (192.0 eV in the B 1*s* spectrum) besides these organic components (*e.g.*, $ROCO_2Li$). These featured F- and B-containing species originates from $DFOB^-$ anion in the CIP mode (Fig. 2c). Although this SEI chemistry of LiDFOB-FDMA electrolyte contains the reduction product of $DFOB^-$ due to the formation of anion-participated solvation, the undesirable component cannot provide the persistent stability toward Li metal (Fig. S10).

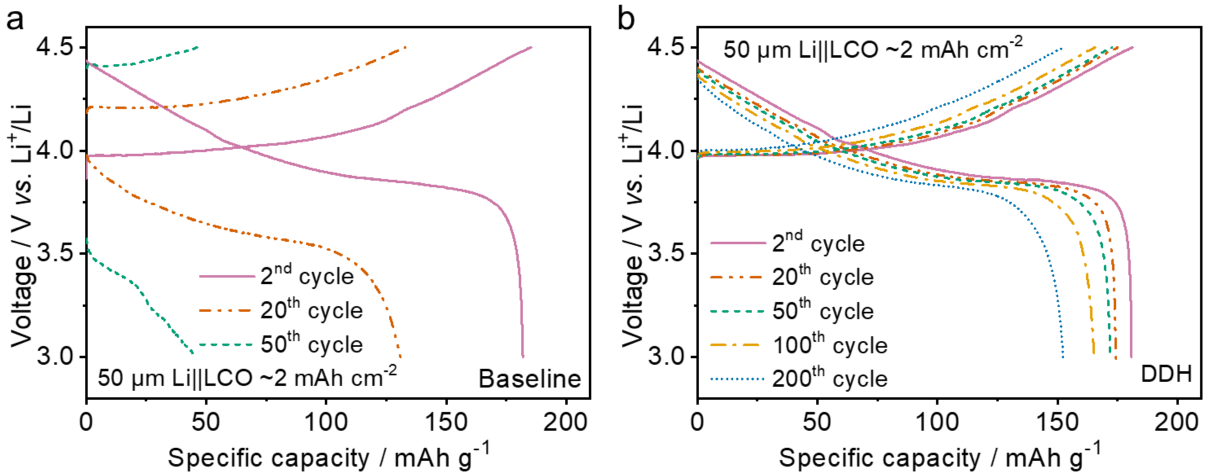


**Fig. S20** Charge-discharge voltage profiles of $Li||LiCoO_2$ cells employing **a** baseline and **b** DDH electrolytes at different cycles

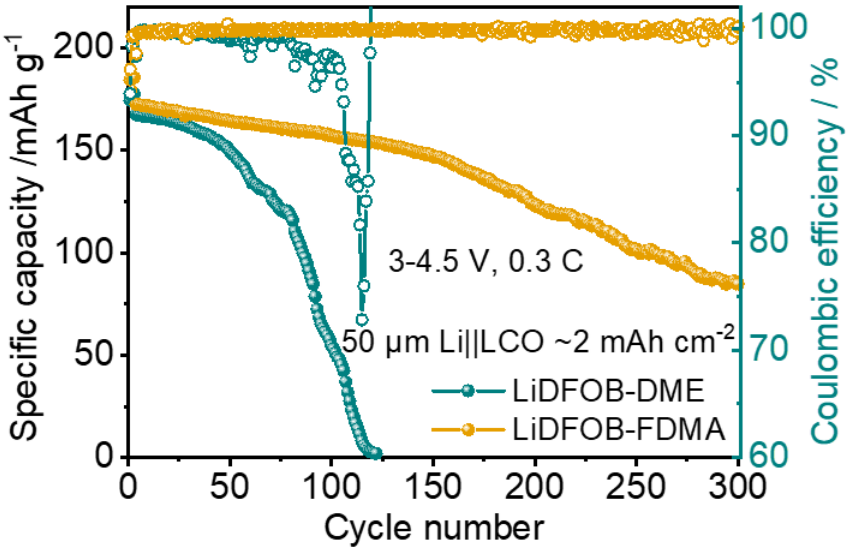


**Fig. S21** Cycling performance of Li||$LiCoO_2$ cells using LiDFOB-DME and LiDFOB-FDMA electrolytes

It can be seen that the Li||$LiCoO_2$ cells with LiDFOB-DME electrolyte show a sharp drop in capacity after 50 cycles due to the poor oxidation stability of DME solvent, resulting in the continuous decomposition of the electrolyte and consequently to the deterioration of battery performance [S7]. While those using LiDFOB-FDMA electrolyte deliver 49% capacity retention at the 300$^{th}$ cycle. Although the FDMA solvent with weak solvation ability with $Li^+$ form CIP solvation sheath (Fig. 2c), the SEI component formed in LiDFOB-FDMA electrolyte is inadequate to maintain a stable long-term cycling of batteries.

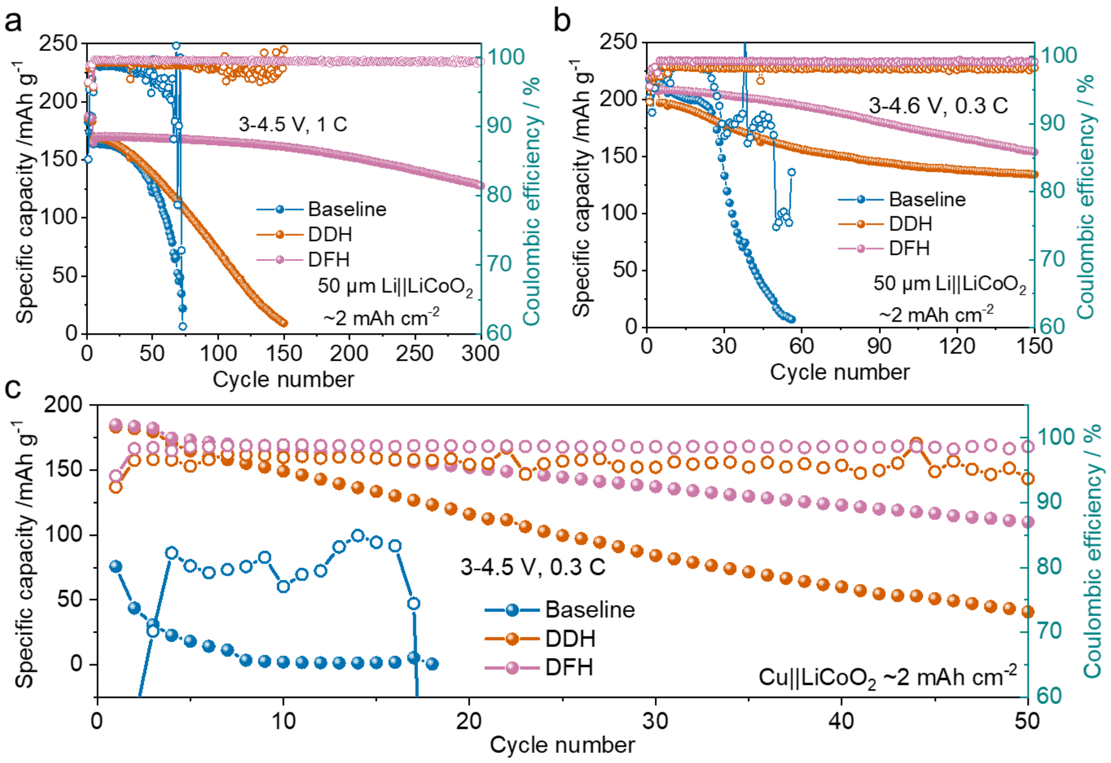


**Fig. S22** Long-term cycling performance of Li||$LiCoO_2$ cells using different electrolytes **a** under a voltage range of 3-4.5 $V_{Li}$ at 1 C, **b** under a voltage range of 3-4.6 $V_{Li}$ at 0.3 C, **c** with Li-free under a voltage range of 3-4.5 $V_{Li}$ at 0.3 C

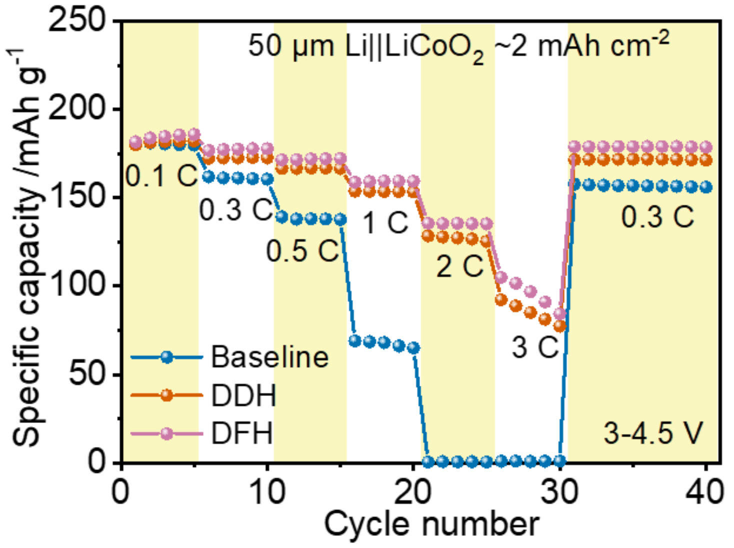


**Fig. S23** Rate performances of Li||$LiCoO_2$ cells using different electrolytes under a voltage range of 3-4.5 $V_{Li}$

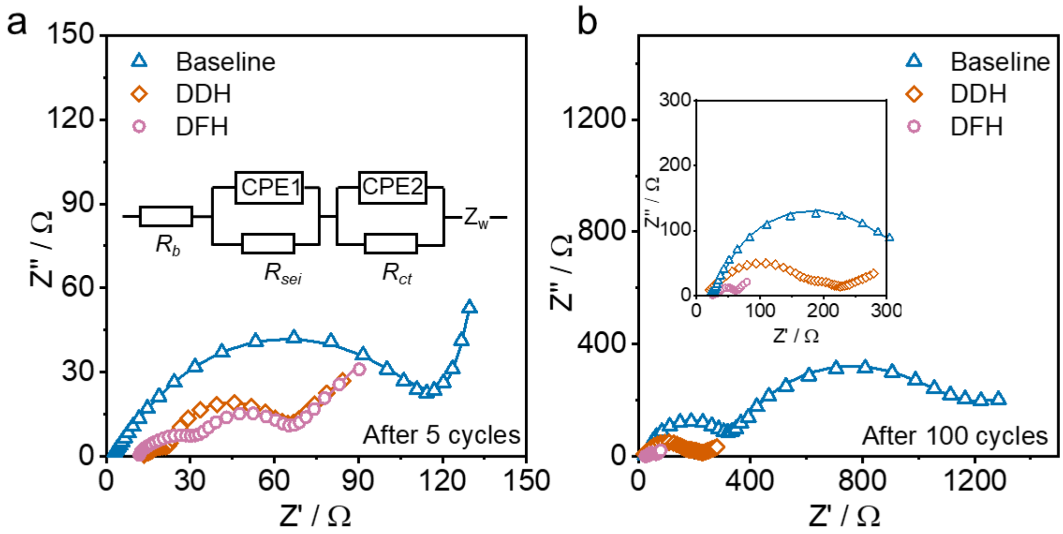


**Fig. S24** EIS spectra of the Li||$LiCoO_2$ cells using four electrolyte samples after **a** 5 cycles and **b** 100 cycles at 100 mA $g^{-1}$O 1*s* in-depth XPS spectra of the Cu substrate obtained from the Li||Cu cells using baseline electrolyte. LiF: 685 eV [S8]; $P_xO_yF_z$: 687 eV [S9]

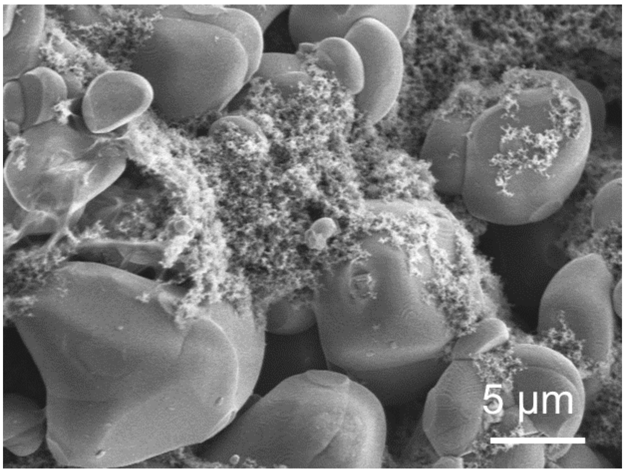


**Fig. S25** FE-SEM image of pristine $LiCoO_2$ electrode

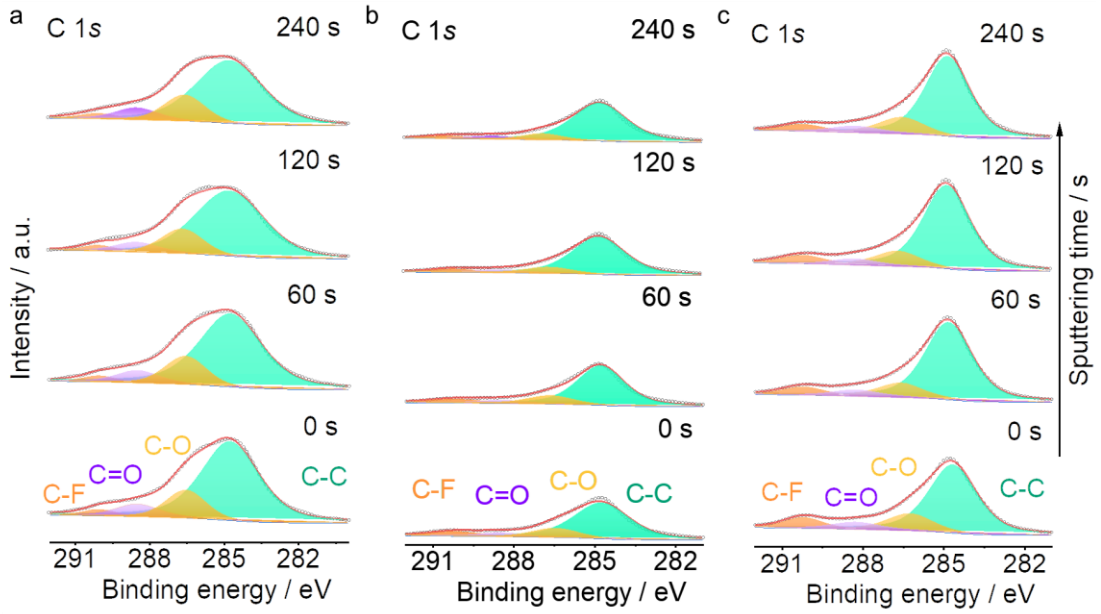


**Fig. S26** C 1*s* XPS in-depth spectra of the $LiCoO_2$ cathodes obtained from the cycle Li||$LiCoO_2$ cells using **a** baseline, **b** DDH, and **c** DFH electrolyte after 50 cycle at 0.3C. C-C: 284.8 eV, C-O: 286.5 eV; C=O:288 eV; C-F: 290 eV [S1]

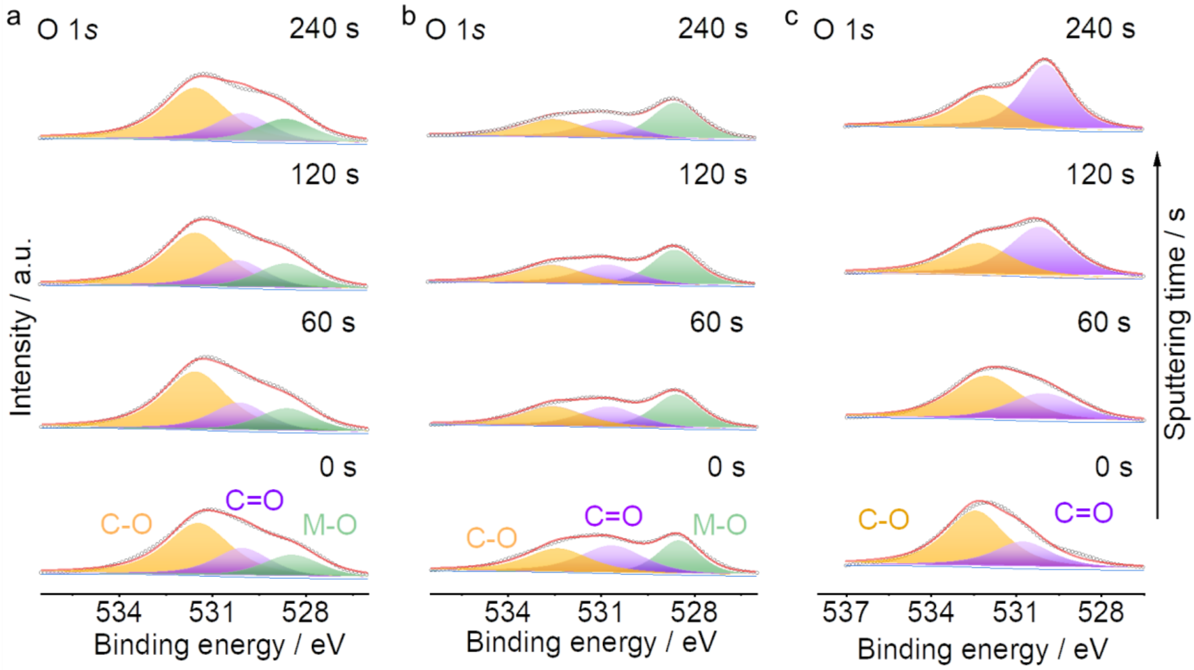


**Fig. S27** O 1*s* XPS in-depth spectra of the $LiCoO_2$ cathodes obtained from the cycle Li||$LiCoO_2$ cells using **a** baseline, **b** DDH, and **c** DFH electrolyte after 50 cycle at 0.3C. M-O: 528.5 eV [S5]; C=O: 531.5 eV [S2]; C-O=533 eV [S3]

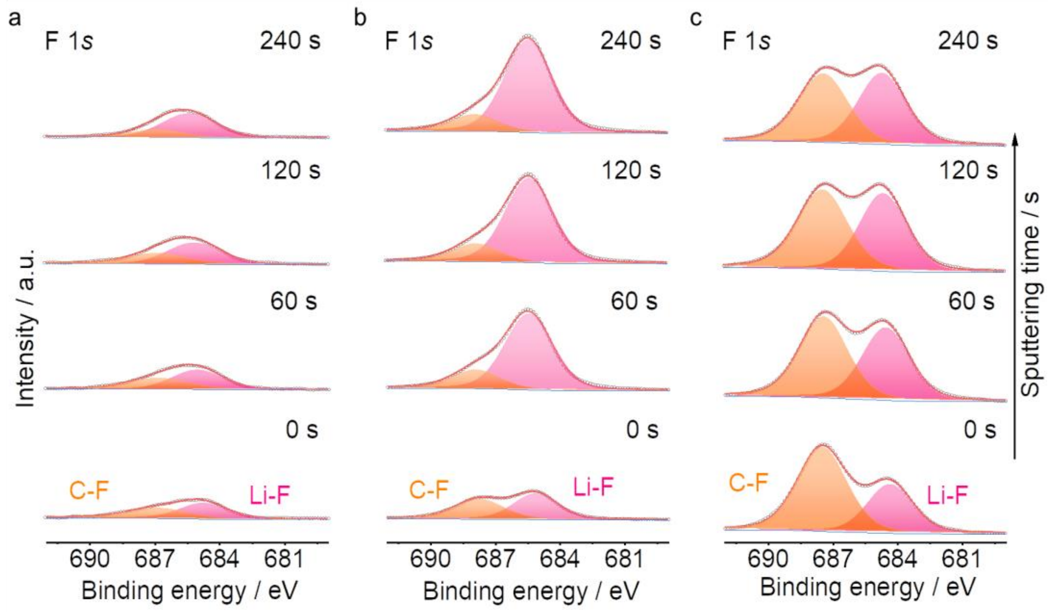


**Fig. S28** F 1*s* XPS in-depth spectra of the $LiCoO_2$ cathodes obtained from the cycle Li||$LiCoO_2$ cells using **a** baseline, **b** DDH, and **c** DFH electrolyte after 50 cycle at 0.3C. LiF: 685 eV; C-F: 688 eV [S6]

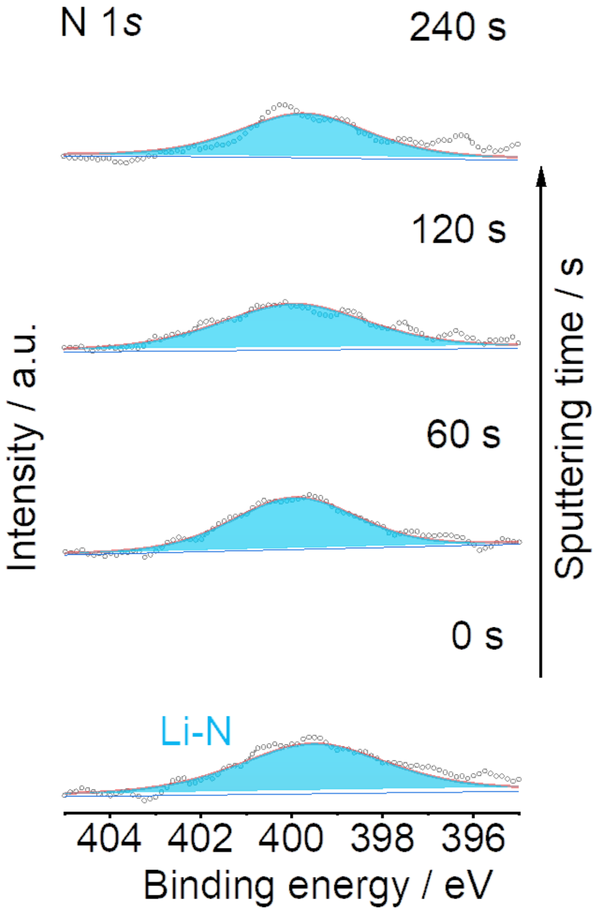


**Fig. S29** N 1*s* XPS in-depth spectra of the $LiCoO_2$ cathodes obtained from the cycle Li||$LiCoO_2$ cells using DFH electrolyte after 50 cycle at 0.3C. $Li_3N$: 399.0 eV [S10]

**Table S1** Fitted values of the impedance spectra in Li||Li cells using baseline electrolyte (Fig. S9b)

| T (K) | 1000/T (1/K$^{-1}$) | $R_{sei}$ | $R_{ct}$ | Ln(T/$R_{sei}$) | Ln(T/$R_{ct}$) |
|---|---|---|---|---|---|
| 283 | 3.533569 | 32.7 | 89.45 | 2.158072 | 1.151767 |
| 293 | 3.412969 | 10.19 | 47.48 | 3.358766 | 1.819864 |
| 303 | 3.30033 | 5.832 | 19.33 | 3.950373 | 2.752075 |
| 313 | 3.194888 | 3.03 | 9.322 | 4.637641 | 3.513826 |
| 323 | 3.095975 | 1.1 | 4.58 | 5.682342 | 4.255953 |

**Table S2** Fitted values of the impedance spectra in Li||Li cells using DDH electrolyte (Fig. S9c)

| T (K) | 1000/T (1/K$^{-1}$) | $R_{sei}$ | $R_{ct}$ | Ln(T/$R_{sei}$) | Ln(T/$R_{ct}$) |
|---|---|---|---|---|---|
| 283 | 3.533569 | 3.833 | 73.04 | 3.833 | 1.35444 |
| 293 | 3.412969 | 2.738 | 37.34 | 2.738 | 2.060107 |
| 303 | 3.30033 | 1.633 | 16.46 | 1.633 | 2.9128 |
| 313 | 3.194888 | 1.069 | 6.777 | 1.069 | 3.832669 |
| 323 | 3.095975 | 0.699 | 3.88 | 0.699 | 4.421817 |

**Table S3** Fitted values of the impedance spectra in Li||Li cells using DFH electrolyte. (Fig. S9d)

| T (K) | 1000/T (1/K$^{-1}$) | $R_{sei}$ | $R_{ct}$ | Ln(T/$R_{sei}$) | Ln(T/$R_{ct}$) |
|---|---|---|---|---|---|
| 283 | 3.533569 | 4.97 | 60.98 | 4.042027 | 1.534901 |
| 293 | 3.412969 | 3.359 | 33.92 | 4.468529 | 2.156168 |
| 303 | 3.30033 | 1.951 | 14.81 | 5.045391 | 3.01843 |
| 313 | 3.194888 | 1.347 | 6.862 | 5.448323 | 3.820204 |
| 323 | 3.095975 | 1.056 | 3.769 | 5.723164 | 4.450843 |

**Table S4** EIS simulation results of the Li||$LiCoO_2$ cells in the three electrolyte samples. The results are obtained from the three electrolyte samples after selected cycles corresponding to Fig. S24

| Electrolytes | $R_{sei}$ at 5th | $R_{sei}$ at 100th | $R_{ct}$ at 5th | $R_{ct}$ at 100th |
|---|---|---|---|---|
| 1 M LiPF6-EC: EMC | 58.63 | 228.10 | 65.10 | 605.3 |
| DDH | 10.98 | 141.80 | 33.37 | 90.58 |
| DFH | 25.47 | 13.63 | 22.30 | 19.52 |

## Supplementary References

[S1] M. He, C.C. Su, Z. Feng, L. Zeng, T. Wu et al., High voltage $LiNi_{0.5}Mn_{0.3}Co_{0.2}O_2$/graphite cell cycled at 4.6 V with a FEC/HFDEC-based electrolyte. Adv. Energy Mater. **7**(15), 1700109 (2017).

https://doi.org/10.1002/aenm.201700109

[S2] X. Fan, L. Chen, O. Borodin, X. Ji, J. Chen et al., Non-flammable electrolyte enables Li-metal batteries with aggressive cathode chemistries. Nat. Nanotechnol. **13**(8), 715-722 (2018). https://doi.org/10.1038/s41565-018-0183-2

[S3] Y. Zhang, Y. Zhong, Z. Wu, B. Wang, S. Liang et al., Solvent molecule cooperation enhancing lithium metal battery performance at both electrodes. Angew. Chem. Int. Ed. **59**(20), 7797-7802 (2020). https://doi.org/10.1002/anie.202000023

[S4] J. Xia, L. Madec, L. Ma, L.D. Ellis, W. Qiu et al., Study of triallyl phosphate as an electrolyte additive for high voltage lithium-ion cells. J. Power Sources **295**, 203-211 (2015). https://doi.org/10.1016/j.jpowsour.2015.06.151

[S5] S. Jiao, X. Ren, R. Cao, M.H. Engelhard, Y. Liu et al., Stable cycling of high-voltage lithium metal batteries in ether electrolytes. Nat. Energy **3**(9), 739-746 (2018). https://doi.org/10.1038/s41560-018-0199-8

[S6] J. Wu, X. Wang, Q. Liu, S. Wang, D. Zhou et al., A synergistic exploitation to produce high-voltage quasi-solid-state lithium metal batteries. Nat. Commun. **12**, 5746 (2021). https://doi.org/10.1038/s41467-021-26073-6

[S7] M. Li, C. Wang, Z. Chen, K. Xu, J. Lu, New concepts in electrolytes. Chem. Rev. **120**(14), 6783–6819 (2020). https://doi.org/10.1021/acs.chemrev.9b00531

[S8] L.L. Jiang, C. Yan, Y.X. Yao, W. Cai, J.Q. Huang et al., Inhibiting solvent co-intercalation in a graphite anode by a localized high-concentration electrolyte in fast-charging batteries. Angew. Chem. Int. Ed. **60**(7), 3402-3406 (2020). https://doi.org/10.1002/anie.202009738

[S9] W.H. Li, Q.L. Ning, X.T. Xi, B.H. Hou, J.Z. Guo et al., Highly improved cycling stability of anion de-/intercalation in the graphite cathode for dual-ion batteries. Adv. Mater. **31**(4), 1804766 (2019). https://doi.org/10.1002/adma.201804766

[S10] X. Wang, S. Wang, H. Wang, W. Tu, Y. Zhao et al., Hybrid electrolyte with dual-anion-aggregated solvation sheath for stabilizing high-voltage lithium-metal batteries. Adv. Mater. **33**(52), 2007945 (2021). https://doi.org/10.1002/adma.202007945
